# Community Assembly Reveals How Environmental Controls Over Rodent Competition Drive Deer Mouse Density and Hantavirus Infection

**DOI:** 10.1111/ele.70374

**Published:** 2026-03-30

**Authors:** Angela D. Luis, Dean E. Pearson

**Affiliations:** ^1^ Department of Ecosystem and Conservation Sciences University of Montana Missoula Montana USA; ^2^ Division of Biological Sciences University of Montana Missoula Montana USA; ^3^ Rocky Mountain Research Station, USDA Forest Service Forestry Sciences Laboratory Missoula Montana USA

**Keywords:** community disassembly, dilution effect, diversity‐disease relationships, hantavirus, resource competition, sin Nombre virus, substitutive assembly

## Abstract

Host diversity can strongly influence disease prevalence, but whether it dilutes or amplifies disease remains debated. We applied community assembly theory to examine whether conditionality from abiotic and biotic filtering could explain variation in rodent diversity and Sin Nombre hantavirus (SNV) prevalence across 24 locations in the southwestern United States. Overall, community composition, not diversity per se, drove diversity‐disease relationships. Environmental factors determined community composition, which regulated primary host abundance and SNV infection via resource competition. Across roughly half the communities, dilution effects emerged because added species increased dietary overlap, reducing focal host abundance and SNV infection. In other communities, environmental and biotic structuring favoured competitors, suppressing host abundance and SNV infection across diversity levels. Our results highlight how environmental structuring and substitutive assembly processes interact to influence diversity‐disease patterns. Community assembly theory provides a framework for integrating abiotic and biotic processes to inform landscape‐scale disease patterns.

## Graphical abstract

We examined how community assembly processes shape Sin Nombre hantavirus (SNV) dynamics in rodent communities across the southwestern United States. Environmental factors structured community composition, which regulated deer mouse abundance and SNV infection, rather than diversity alone. Our results show that host regulation through competition underlies negative diversity–disease relationships, highlighting community assembly as a framework for predicting landscape‐scale disease risk.

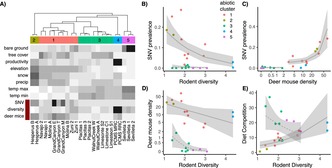

## 1 Introduction

Zoonotic diseases comprise 60% of emerging infectious diseases, posing major threats to human populations (Rahman et al. 2020). Understanding the processes governing zoonotic disease dynamics is essential for predicting and managing outbreaks. While studies have emphasised the role of biotic factors such as host diversity in influencing pathogen transmission (Estrada‐Peña et al. 2014; Khalil et al. 2016; Lafferty and Wood 2013; Ostfeld and Keesing 2000; Shaw and Civitello 2021), abiotic factors like temperature and precipitation also play key roles (Khasnis and Nettleman 2005; Mordecai et al. 2019). Because anthropogenic change is altering both biotic and abiotic conditions (Boivin et al. 2016; Didham et al. 2007; Penuelas et al. 2020), approaches that integrate these drivers are critical for understanding and predicting disease dynamics at large spatial scales.

Biodiversity loss has intensified interest in how species diversity influences pathogen prevalence (Khalil et al. 2016; Lafferty and Wood 2013; Rohr et al. 2020; Shaw and Civitello 2021). It was initially postulated that high diversity would reduce infection prevalence as incompetent host species could reduce transmission among competent hosts (Ostfeld and Keesing 2000). However, studies report positive, negative and neutral effects (Khalil et al. 2016; Luis et al. 2018; Shaw and Civitello 2021; Wood et al. 2014), leading to debate about the functional role that diversity plays in disease ecology (Dobson et al. 2006; Ostfeld and LoGiudice 2003; Wood and Lafferty 2013; Rohr et al. 2020). More recent work emphasises community composition over diversity per se, highlighting how community assembly/disassembly processes—how species are added to or lost from a community—can strongly influence the presence and abundance of competent versus non‐competent hosts (LoGiudice et al. 2003; Rohr et al. 2020). If assembly is substitutive, individuals of new species will replace individuals of former species, whereas additive assembly can result in new species adding individuals to the community. These processes combined with sampling effects (increasing diversity elevates chances of high or low competency hosts, e.g., Halliday et al. 2017), community nestedness (low diversity communities contain a nested subset of higher diversity communities, e.g., Johnson et al. 2013), and directional disassembly (e.g., anthropogenic diversity loss favours weedy species that are more competent hosts, Halliday et al. 2020) can provide mechanistic understandings of how community assembly/disassembly relates to infection prevalence within a specific community. However, this focus on biotic assembly overlooks a key element of community assembly theory: processes are hierarchical, with abiotic filters typically preceding biotic interactions (Keddy 1992; Keddy and Laughlin 2021). Hence, understanding how assembly shapes community composition and pathogen prevalence across communities requires integrating both abiotic and biotic filters.

One plausible explanation for conflicting effects of diversity is that infection dynamics are context dependent, changing across communities as shifting abiotic conditions alter host species composition and, thereby, biotic interactions. Abiotic factors strongly influence disease dynamics (Armon and Cheruti 2012), but their interaction with host abundance and diversity remains poorly understood. Interacting abiotic and biotic processes could plausibly generate the documented range of positive, negative and neutral diversity‐disease relationships, revealing when each pattern arises rather than which is universally correct. Integrating these processes is essential for explaining how ecological context influences biotic interactions and disease processes across landscapes, especially under accelerating anthropogenic change.

Community assembly theory provides a framework for this integration by linking abiotic and biotic filters to species' traits to explain community composition (Keddy 1992; Keddy and Laughlin 2021). Grounded in Hutchinsonian niche theory, it distinguishes between a species' fundamental niche, set by abiotic limits, and the realised niche, constrained by biotic interactions (Hutchinson 1957). These processes can also interact. For example, sublethal physical stress may increase susceptibility to biotic effects (Lomolino et al. 2010), whereas facilitation could allow species to expand beyond their normal physiological limits (Bruno et al. 2003). Applying this framework to disease ecology could integrate abiotic and biotic drivers to explain how variation in community composition influences disease dynamics.

Here, we apply community assembly theory to evaluate how abiotic and biotic processes shape the relationship between rodent community diversity and Sin Nombre hantavirus (SNV) infection in deer mouse ( *Peromyscus maniculatus* ) populations across the southwestern United States. SNV causes hantavirus pulmonary syndrome (HPS), which has ~35% case fatality in humans. This system is ideal for evaluating these questions because (1) a variety of diversity‐prevalence relationships have been documented (Clay et al. 2009; Luis et al. 2018; Vadell et al. 2020) and (2) long‐term U.S. Centers for Disease Control and Prevention (CDC) monitoring across 24 rodent communities provides extensive spatial data (Mills, Yates, et al. 1999).

Deer mice are the primary reservoir for this directly‐transmitted pathogen (Mills, Ksiazek, et al. 1999). The ‘dilution effect’ was originally defined as an encounter‐reduction mechanism, where greater species diversity reduces contacts between vectors and competent hosts, lowering infection risk. The term has since been applied more broadly to any negative diversity‐disease relationship (Keesing et al. 2006; Rohr et al. 2020), creating the need to clarify underlying mechanisms. For directly‐transmitted pathogens, mechanisms include host regulation (e.g., via competition or predation), encounter reduction (e.g., behavioural changes limiting contacts), transmission reduction (e.g., lower per‐contact infection probability), increased mortality of infected hosts (e.g., selective predation) and enhanced recovery (Keesing et al. 2006). Host regulation appears most relevant for SNV: diversity reduces prevalence primarily when it lowers deer mouse density, whereas no effect on density yields no effect on prevalence (Eleftheriou and Luis 2025), underscoring the need to examine how diversity relates to host density as a mechanism influencing SNV dynamics. While most prior SNV studies focused on prevalence (Eleftheriou and Luis 2025), risk to humans is more directly captured by infected host density. We therefore evaluated how abiotic and biotic factors structure rodent communities, influence deer mouse abundance and determine both prevalence and infected host density.

Following community assembly theory, we hypothesised that (1) abiotic and biotic filters hierarchically structure rodent communities, with abiotic factors primarily determining local species composition via fundamental niche requirements; (2) environmental effects on community composition then determine how biotic interactions influence host abundance and diversity‐disease relationships; and (3) species traits mediate the influence of environmental and biotic filtering on community structure and disease outcomes.

## 2 Materials and Methods

### 2.1 Study System

**FIGURE 1:**
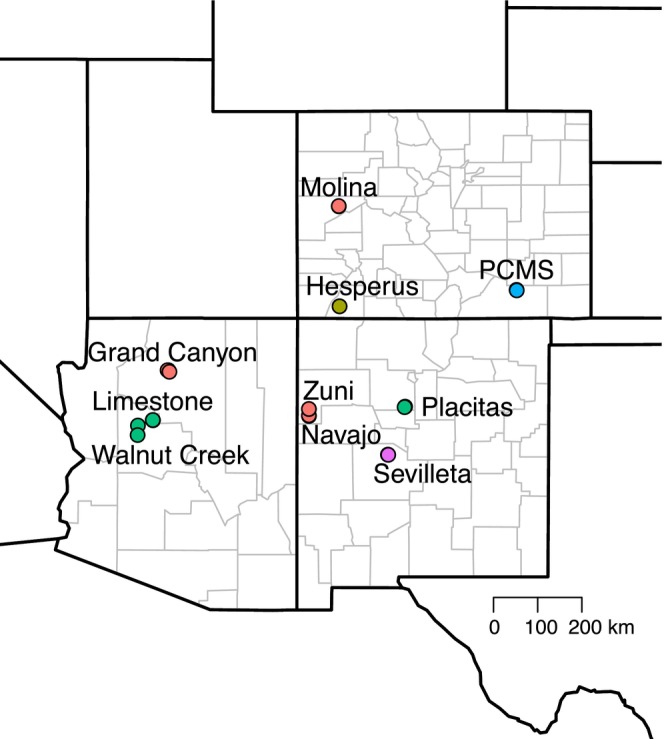
Map of study sites in the southwestern United States. Colours reflect rodent community types as determined by environmental cluster analysis. Each web is plotted individually, but numerous points overlap due to multiple webs within a site converging at this map scale. Only the larger sites, consisting of 2–3 webs, are labelled.

We used a long‐term CDC‐funded rodent monitoring dataset (1994–2006), covering 24 trapping webs across 10 sites (2–3 webs per site) spanning three southwestern U.S. states (Figure 1) (Mills, Yates, et al. 1999), the epicentre for HPS cases. This system enables investigation of how SNV prevalence is shaped by biotic and abiotic processes across a large landscape.

Prior work focused on a subset of these sites (Luis et al. 2018), but here we expand analyses to all 24 webs using traditional and novel approaches to assess broader diversity‐disease patterns. Analyses were performed in R (R Core Team 2023) and data and code are archived on Zenodo (https://doi.org/10.5281/zenodo.18744824).

### 2.2 Data

Each 3.14‐ha web was trapped monthly for three consecutive nights during the growing season (May–September) using Sherman traps. Blood samples were tested for SNV antibodies at the CDC (Atlanta, GA). Because deer mice remain chronically infected with SNV for life (Bagamian et al. 2013), we use ‘prevalence’ to refer to the proportion of deer mice with antibodies. We assumed prevalence was zero if no deer mice were caught at a web in a given month, because excluding zero‐capture months disproportionately inflates mean prevalence at low‐abundance sites, and the absence of the reservoir host corresponds to negligible spillover risk at that web during that period. To capture broadscale patterns reflecting community assembly processes (Keddy and Laughlin 2021), rather than within‐site temporal dynamics, we calculated mean species abundances for all rodent species, mean deer mouse infection prevalence, and mean density of infected deer mice per web during the growing season (from May to September) over the 12‐year study period, adjusting for species‐specific capture rates (Appendix S1 Methods; Figure S1).

Vegetation cover and productivity (30 m annual resolution) were derived from the Rangeland Analysis Platform (Allred et al. 2021; Jones et al. 2021), and climate variables (1 km daily resolution) from Daymet (Thornton et al. 2022) via the *daymetr* package (Hufkens et al. 2018); all variables were averaged over the 12‐year period for each web (Appendix S1 Methods). Phylogenetic distances between deer mice and other rodents were calculated using a mammalian supertree (Bininda‐Emonds et al. 2007) via the *ape* package (Paradis and Schliep 2019). We obtained species data on diet and activity (nocturnal, diurnal, crepuscular) from the EltonTraits 1.0 database (Wilman et al. 2014) and average adult body mass from the Amniote life‐history database (Myhrvold et al. 2015). Trait similarity with deer mice was standardised (0–1; Appendix S1) to reflect potential niche overlap and competitive strength, approximating a Lotka‐Volterra competition coefficient (Gotelli 1998).

### 2.3 Biotic Emphasis for Evaluating Dilution Effects

For comparison with prior work, we first evaluated how small mammal diversity related to SNV risk following Luis et al. (2018), who reported a negative diversity‐prevalence relationship at 9 of the 24 webs. We modelled web‐level mean prevalence with a binomial GLM and mean density of infected deer mice with a Tweedie GLM fit via the *glmmTMB* function (McGillycuddy et al. 2025) with inverse Simpson's diversity metric as the predictor, which accommodates continuous responses (due to averaging species abundances across years) and zeroes. This allowed us to test whether the previously observed dilution effect extended to the additional 15 webs in our dataset (Appendix S1 for details, diagnostics and CI estimation).

### 2.4 Partitioning Environmental and Biotic Effects on Community Composition

To evaluate the relative roles of environmental versus biotic factors in structuring rodent communities (H_1_), we applied redundancy analysis (RDA; Borcard et al. 2018) via the *vegan* package (Oksanen et al. 2022). RDA partitions variation in species abundances into components explained by environmental variables (constrained) and residual variation (unconstrained), which may reflect biotic (interspecific) interactions. The web × species response matrix was Hellinger‐transformed (Legendre and Gallagher 2001) using the *decostand* function. Predictor variables included min/max temperature, precipitation, snow water equivalent, elevation, productivity, percent tree cover and percent bare ground.

### 2.5 Biotic Effects on Host Abundance and Linkages to Traits

We evaluated how biotic interactions influenced deer mouse (*pm*) abundance, considering species traits—phylogenetic relatedness, body size, nocturnality, diet overlap (H_2_, H_3_). Abundance was modelled using Tweedie GLMs to accommodate non‐negative continuous data with zeros, with the variance power parameter estimated by profile likelihood (*p* = 1.83) and held constant across models for valid AIC comparison (Appendix S1).

Each regression took the form, *pm*_*j*_ = *β*_0_ *+ β*_1_ *·*∑_*i*_ *α*_*i*_ *N*_*i,j*_, where *N*_*i,j*_ is the abundance of species *i* (excluding deer mice) at web *j*, and *α*_*i*_ is similarity between species *i* and deer mice, based on each trait. We ran four independent models, each with a different *α*: (1) phylogenetic similarity, (2) dietary overlap, (3) body mass similarity and (4) nocturnality (1 for nocturnal species, 0 otherwise). Each model estimated two parameters, *β*_0_ (intercept) and *β*_1_ (slope). Here, *α*_*i*_ *β*_1_ is equivalent to a Lotka‐Volterra competition coefficient for species *i* (based solely on the examined similarity in phylogeny or trait). Therefore, ∑_*i*_ *α*_*i*_ *N*_*i,j*_ represents the total competitive pressure on deer mice over all rodent species based on niche overlap of that trait. For comparison, we also tested a model using inverse Simpson's diversity index as the predictor. We compared the five models by AIC.

Lotka‐Volterra theory predicts that competition interacts with environmental constraints such that equilibrium abundance at a site depends on both the strength of interspecific competition and the site's carrying capacity (Gotelli 1998). To explore whether variation in potential resource availability could improve model fit, we assessed whether adding productivity (Appendix S1) to the best competition model was supported by AIC.

We used web as the replicate because there were some differences in rodent community composition and environmental variables across webs within a site. Our inference focuses on explaining broad‐scale variation among sites; including site as a random effect would emphasise within‐site contrasts and absorb the between‐site variation of interest. To assess potential pseudoreplication, we repeated all regressions at the site level, averaging across webs (see Appendix S1).

### 2.6 Community Nestedness

To evaluate whether patterns of community structuring could help explain diversity–disease relationships (H1, H2), we quantified nestedness and species turnover across webs using presence–absence matrices. Total beta diversity (Sørensen dissimilarity, β_SOR_) was partitioned into turnover (β_SIM_) and nestedness‐resultant (β_SNE_) components using the *beta.multi* function from the *betapart* R package (Baselga et al. 2023). Nestedness was assessed using matrix temperature (Rodríguez‐Gironés and Santamaría 2006) via *vegan*'s *nestedtemp* function (Oksanen et al. 2022), tested against null models using *oecosimu* with the “quasiswap” algorithm, which preserves species incidence and site richness. We also calculated the NODF (Nestedness metric based on Overlap and Decreasing Fill) index using the *nestednodf* function and tested against the same null model (Almeida‐Neto et al. 2008). Co‐occurrence patterns were evaluated using the *nestedchecker* function, which calculates the C‐score, a metric of species segregation based on checkerboard units (Stone and Roberts 1990). These analyses provided insight into whether low‐diversity communities reflected subsets of higher‐diversity ones or assembled through distinct species replacement, which could mediate diversity–disease patterns.

### 2.7 Structural Equation Models

To integrate the multiple interacting pathways identified above, we used structural equation models (SEMs) to synthesise relationships among key driver and response metrics—abiotic factors, competitor communities, deer mouse abundance and SNV risk and evaluate if abiotic factors affect deer mouse abundance directly or indirectly through competitor abundance. Abiotic variables (temperature, precipitation, snow water equivalent and elevation) were summarised using principal components analysis (PCA), and the first three components were summed into a single abiotic index based on loadings. For biotic interactions, we included the covariate(s) receiving the strongest support from the trait‐based regressions (above).

To accommodate bounded variables with zeroes while preserving causal structure, prevalence and density measures were transformed prior to SEM analysis (Appendix S1 Methods); distributionally explicit generalised linear models were used for primary inference. We tested competing a priori hypotheses regarding whether abiotic conditions primarily structured competitor communities rather than directly affecting deer mice, and whether Simpson's diversity index explained additional variation. All variables were standardised (mean = 0, SD = 1). The SEMs were fit using the lavaan package (Rosseel 2012), evaluated with standard fit indices, and compared using AIC.

### 2.8 Community Structuring and the Dilution Effect

We applied cluster analyses as a post hoc tool for visualising how environmental filtering shapes community structure and mediates diversity‐disease relationships (H1, H2, H3). This approach allowed us to assign rodent communities to distinct community types based on environmental factors, thereby mirroring the results from the RDA but overcoming the RDA constraint to continuous output. We assigned rodent assemblages to unique community types based on similarity of underlying environmental factors. First, we calculated the Euclidean distance between each web based on environmental conditions to create a dissimilarity matrix, using the *dist* function on the standardised environmental variable matrix. We compared several hierarchical clustering algorithms (Appendix S1 and Borcard et al. (2018)). The number of clusters (*k* = 5) was chosen based on multiple optimisation criteria and biological interpretability (Appendix S1). Following clustering, we used post hoc GLMs to assess mechanisms underlying the dilution effect by grouping clusters exhibiting dilution and analysing associations among diversity, host density, competitive traits and SNV infection.

## 3 Results

The dataset spans > 247,000 km^2^ (by minimum convex polygon) across 3 southwestern US states (Figure 1). Across the 24 webs, 34 rodent species were recorded (6–15 per web). Average deer mouse densities varied from 0 to 66 per web. Webs experienced a range of climatic conditions, including average annual temperatures from 7.7°C to 14.3°C, average annual precipitation from 225.8 to 555.4 mm and elevations from 1433 to 2395 m.

### 3.1 Biotic Emphasis for Evaluating Dilution Effects

**FIGURE 2:**
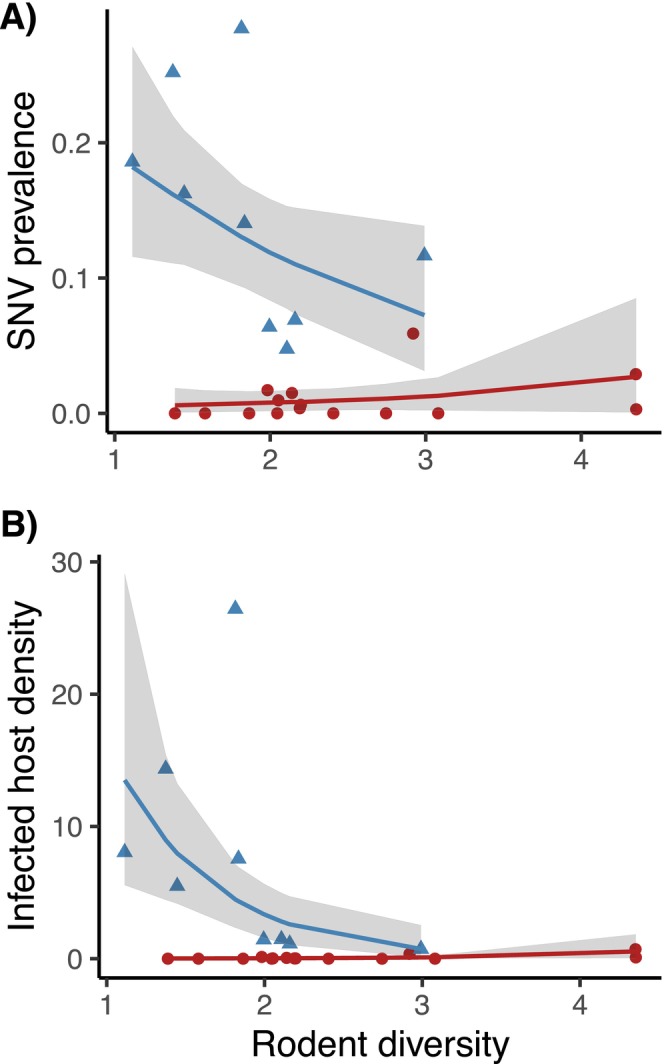
A traditional approach to analysing diversity‐disease relationships that emphasises biotic interactions (diversity) following Luis et al. (2018). The figure shows the relationship between average rodent diversity (inverse Simpson's Diversity index) and average SNV prevalence (A) or average density of SNV‐infected deer mice per site (B) for 24 webs where CDC‐funded researchers monitored SNV from 1994 to 2006 in the southwestern United States. Webs included in Luis et al. (2018) represented with blue triangles have a significant negative relationship, which is absent in the additional webs included here, represented with red circles. These results provide a baseline for applying community assembly approaches to understand what factors drive such divergence in diversity‐disease patterns.

The significant negative correlation between rodent diversity and SNV prevalence documented at nine webs by Luis et al. (2018) was not observed in the 15 webs added to the dataset in this study (Figure 2a blue vs. red; Table S2). Patterns were consistent when using density of infected hosts as the response (Figure 2b; Table S3). This result motivates a community assembly approach to explore why some communities exhibit a dilution effect while others do not.

### 3.2 Partitioning Environmental and Biotic Effects on Community Composition

**FIGURE 3:**
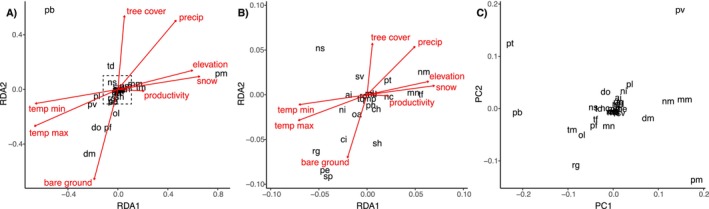
Redundancy Analysis (RDA) results for all rodent species abundances. Both panels (A) and (B) show the first two constrained axes, which together explain 57% of the variation in the rodent community (the first five canonical axes were significant, explaining 76% of variation in the rodent species composition and abundances), driven by the environmental variables indicated in red (Panel B is zoomed into the area of the dashed box in panel A). (C) The first two unconstrained axes, PC1 and PC2, represent how the species are correlated to each other after accounting for the environmental variables. These axes combined explain approximately 9% additional variation. See Appendix Table S1 for species names corresponding to the two‐letter abbreviations in black, for example, pm represents *Peromyscus maniculatus* .

The RDA indicated that the environmental variables—min/max temperature, precipitation, snow water equivalent, elevation, productivity, percent tree cover and percent bare ground, which comprised the constrained axes in the analysis—were all significant predictors (*p <* 0.05, Tables S4 and S5) and collectively explained ~76% of variation (adjusted *R* ^2^) in rodent species composition and abundances between webs. The first 5 canonical axes were significant (Table S6); the first two axes are plotted in Figure 3a,b. The first two unconstrained axes explained an additional 6.3% and 2.6% of variation, likely reflecting biotic (interspecific) interactions (Figure 3c, Table S4).

Abiotic variables filtered taxa according to environmentally adapted traits such that species specialised for desert conditions like granivorous kangaroo rats (*Dipodomys* spp.) and pocket mice (*Perognathus* spp.) were strongly linked to hotter, drier habitats with low vegetation cover and high bare ground (Figure 3a), whereas herbivorous voles (*Microtus* spp.) and omnivorous mice and rats (Neotominae) tended toward wetter habitats with higher herbaceous and tree cover (Figure 3b; except *P. leucopus* which occurred in somewhat hotter, drier sites). Deer mice favoured the wettest sites with higher precipitation and snow‐water equivalent (Figure 3a), consistent with their broader distribution that extends farther north into colder, wetter realms than most other rodents. Many other species clustered at the centroid for intermediate environmental values, suggesting these conditions favoured higher rodent diversity and density.

### 3.3 Biotic Effects on Host Abundance and Linkages to Traits

We used GLMs to assess how interspecific competition, based on phylogenetic and functional trait similarity, influenced deer mouse abundance. Dietary overlap received the strongest AIC support (Table S7; Figure S3), suggesting resource competition. Adding productivity to this model substantially improved fit and captured all AIC weight (Table S7). Summed rodent abundances weighted by their dietary overlap had a negative effect on deer mouse abundance and productivity had a positive effect (Table S8), suggesting higher productivity may buffer competitive effects. Site‐level analyses showed consistent patterns (Tables S9 and S10).

### 3.4 Community Nestedness

Rodent communities showed high between‐site variation (*β*_SOR_ = 0.86), driven primarily by species turnover (*β*_SIM_ = 0.80) rather than nestedness (*β*_SNE_ = 0.06), indicating that communities were distinct due to species replacement rather than ordered species loss (Baselga 2010). Consistent with this, nestedness metrics showed no significant nested structure (Table S11, Figure S4), suggesting sites with lower diversity were not subsets of more diverse communities but instead hosted distinct assemblages. NODF and C‐scores indicated significant anti‐nestedness (less nesting than random expectation; Table S11).

### 3.5 Structural Equation Models

**FIGURE 4:**
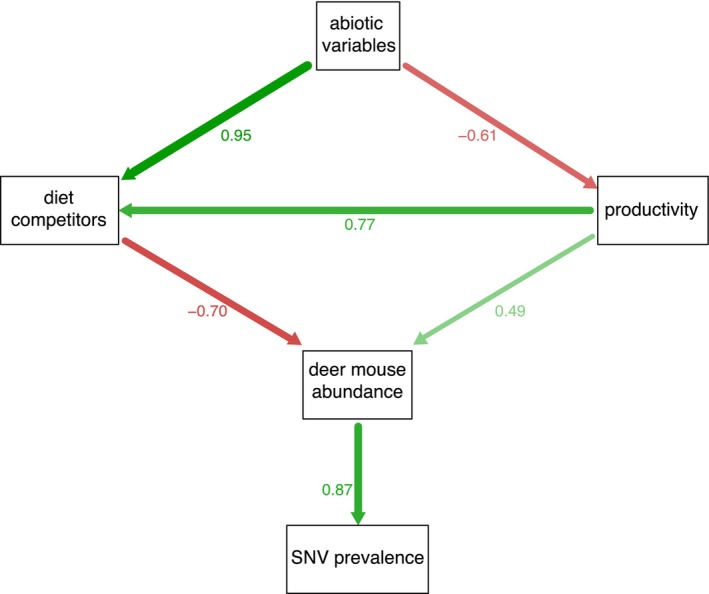
Diagram of fitted structural equation model showing standardised path coefficients (Table S12). The primary driver of SNV prevalence was deer mouse abundance, which was driven by their competitors, with primary productivity mitigating their effects. Abiotic variables determined competitor composition and productivity. There was no support for direct effects of abiotic variables on deer mouse abundance (Table S13).

Since both environmental variables and species interactions were individually supported in separate analyses, we used structural equation modelling (SEM) to understand how they fit together to determine deer mouse abundance and SNV prevalence (Figure 4). We report fully standardised path coefficients (*β*). The best‐fitting SEM (Figure 4; Table S12) included indirect effects of abiotic conditions on deer mouse abundance, mediated through competitor abundance (*β* = 0.95 for the effect of abiotic conditions on competitors; *β* = −0.70 for the effect of competitors on deer mice). Productivity also played a central role: it was influenced by abiotic conditions (*β* = −0.61) and, in turn, positively affected both competitors (*β* = 0.76) and deer mouse abundance (*β* = 0.49). A direct effect of abiotic variables on deer mouse abundance was not supported (*β* = −0.001; *p* > 0.99; Table S13), indicating that environmental conditions shape deer mouse populations indirectly via their effect on community composition and productivity. Deer mouse abundance had a strong positive effect on SNV prevalence (*β* = 0.87), while additional paths involving rodent diversity (Simpson's index) were not supported (Table S14). The overall fit of the final SEM was adequate (*χ* ^2^ = 6.55, df = 4, *p* = 0.161, CFI = 0.97, SRMR = 0.064), though the RMSEA was elevated (0.163), which is expected for small‐sample SEMs with few degrees of freedom. Despite this, all hypothesised paths were strongly supported, directionally consistent with biological expectations, and explained substantial variance in intermediate and response variables: 61% of the variance in competitors, 59% in deer mouse abundance, and 76% in SNV prevalence. Using density of infected deer mice yielded the same conclusions (Appendix S1).

### 3.6 Community Structuring and the Dilution Effect

**FIGURE 5:**
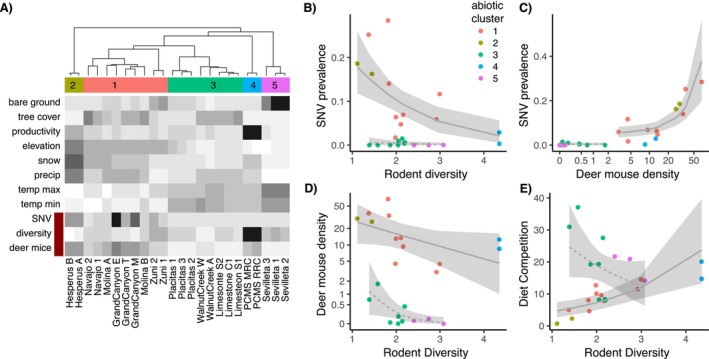
Post hoc investigation using cluster analyses revealed 5 distinct clusters or community types based on abiotic conditions. (A) (Top) dendrogram of hierarchical clustering by environmental variables. Dendrogram tips are coloured by assigned environmental cluster. Beneath the dendrogram, shading shows relative values (darker = higher value) of environmental variables (rows) by site (columns). Rows indicated with the red bar (SNV prevalence, rodent diversity and deer mouse density) were not included in the clustering algorithm but are shown for comparison. To better understand what mediates a dilution effect, we applied a post hoc analysis that grouped clusters by dilution (groups 1, 2 and 4; solid line, B) and non‐dilution responses (groups 3 and 5; dashed line, B) and further analysed community relationships. (B) Relationship between mean rodent diversity by Simpson's D and mean SNV prevalence in deer mice, (C) deer mouse density and SNV prevalence and (D) rodent diversity and deer mouse density, (E) diversity on potential diet competition (the sum of competitor densities times their percent diet overlap with deer mice). Note some axes are on the log scale. See SI Appendix for statistics. Contrasting these results with those from Figure 2 illustrates how applying community assembly approaches to hierarchically account for abiotic and biotic factors can help elucidate drivers of diversity‐disease relationships.

Cluster analyses revealed five environmental clusters reflecting distinct rodent communities (Figures 1, 5a and S5). In post hoc analyses, we examined how SNV prevalence varied with rodent diversity across clusters. Rather than interpreting patterns within individual clusters—which in some cases contained few webs—we evaluated broader groupings of webs that exhibited similar diversity–prevalence relationships. Webs in clusters 1, 2 and 4 collectively showed a negative association between rodent diversity and SNV prevalence, such that webs with lower diversity tended to have higher prevalence (hereafter the ‘dilution group’; Figure 5b, solid line). In contrast, webs from clusters 3 and 5 showed consistently low SNV prevalence regardless of diversity (the ‘non‐dilution group’; Figure 5b, dashed line). Webs previously analysed by Luis et al. (2018) (Figure 2, blue) fell within clusters 1 and 2 and were therefore part of the dilution group. Model comparison using AIC further supported inclusion of cluster 4 in the dilution group (Tables S15 and S16). These post hoc groupings are intended to facilitate biological interpretation of observed patterns and are not presented as independent tests of the dilution hypothesis.

Both groups exhibited high beta diversity driven by species turnover, with low C‐scores indicating anti‐nestedness (Tables S17 and S18; Figures S6 and S7), suggesting sites hosted distinct assemblages. We hypothesised that species identity—especially deer mouse abundance and competitor dominance—might explain differences in SNV patterns. Across all sites, when deer mouse density fell below a threshold, SNV was nearly absent (Figure 5c; Table S19), and all webs in the non‐dilution clusters (3 and 5) had deer mouse densities below this threshold. Notably, these clusters exhibited low deer mouse densities despite relatively low overall rodent diversity (Figure 5d), suggesting that factors other than diversity per se were constraining deer mouse populations. To evaluate these constraints, we examined relationships among deer mouse density, rodent diversity and dietary overlap. Although deer mouse density generally declined with increasing diversity, the form of this relationship differed between dilution and non‐dilution groups (Figure 5d, Table S20). In the dilution group, increasing diversity was associated with greater dietary competition (Figure 5e, dashed line; Table S21), consistent with diversity‐driven suppression of deer mouse density. In contrast, non‐dilution clusters exhibited high dietary overlap even at low diversity (Figure 5e, solid line), indicating strong competitive pressure at low levels of diversity. This elevated diet competition likely suppressed deer mouse densities (Figure 5d, solid line) and, consequently, maintained low SNV prevalence (Figure 5c, solid line).

Community composition played a key role. In the dilution clusters (1, 2, 4), deer mice were generally dominant at the cluster level, though not always at individual sites. Cluster 1 (9 webs) was low in productivity and cool; cluster 2 (2 webs) had moderate productivity, low temperatures and high snow cover; and cluster 4 (2 webs) was highly productive. Despite this variation, deer mouse densities in these clusters typically exceeded the SNV transmission threshold (Figure 5c). In contrast, clusters 3 (8 webs) and 5 (3 webs) showed low deer mouse dominance and density and low SNV transmission. Cluster 3 had low productivity and little snow cover; cluster 5 had high temperatures, low productivity and high bare ground cover. Other species—for example, *P. boylii* , *Perognathus flavus* and *T. dorsalis* in cluster 3; *Dipodomys merriami* , *D. ordii* and *P. flavescens* in cluster 5—were more abundant and likely strong competitors. Even where deer mice were absent from individual webs, they were detected elsewhere on the same site, suggesting competitive exclusion rather than environmental constraints. Using infected mouse density yielded the same conclusions (Figure S8).

## 4 Discussion

Disease ecology has increasingly incorporated community assembly concepts to explain diversity‐disease relationships, but most approaches have emphasised biotic interactions. Our study demonstrates that integrating abiotic and biotic processes within a community assembly framework clarifies why diversity‐disease relationships diverge across landscapes (Figures 1 and 5b,d). Environmental factors were key in rodent community structuring, explaining 76% of overall variation in composition. Deer mice, however, tolerate a broad range of environmental conditions across North America, so abiotic variation rarely constrains them directly. Instead, environmental filtering shapes the competitor community, which in turn regulates deer mouse abundance. Consequently, community composition, not diversity per se, determined how diversity related to disease outcomes. By partitioning biotic interactions from environmental effects, we found that substitutive assembly processes, wherein adding species reduced focal host density, generated both dilution effects and no diversity‐disease relationships, depending on environmental context. Across roughly half of the 24 webs, environmental conditions favoured strong competitors (e.g., *P. truei* and *P. boylii* ) that consistently suppressed deer mouse abundance (Figure 5d dashed line) and thereby SNV prevalence (Figure 5b dashed line) and infected host abundance (Figure S8b) at all diversity levels. In the remaining sites, environmental conditions favoured deer mice, resulting in high deer mouse densities at low diversities. As diversity increased, added species elevated competitive pressure, reducing deer mouse abundance and translating to lower disease risk (Figures 5b and S8b solid lines). These findings emphasise that diversity–disease relationships may depend on environmentally mediated assembly processes governing host–competitor interactions.

The key mechanism underlying the substitutive assembly processes appeared to be resource competition, as supported by our trait‐based analyses (Figure 5e). Dietary overlap exerted a stronger influence on deer mouse suppression than other traits, suggesting that resource competition provides a mechanistic explanation for these patterns. The strong, nonlinear relationship between deer mouse abundance and SNV prevalence (Figure 5c) underscores how modest shifts in host abundance—in this case driven by biotic interactions—can translate into major changes in pathogen dynamics. Structural equation models supported the hypothesis that these biotic interactions were driven by hierarchical filtering, suggesting that abiotic factors largely influenced deer mouse abundance indirectly, through their effects on competitor communities. Together, these results demonstrate that environmental filtering establishes the community template upon which competition acts, jointly determining whether deer mouse populations exceed density thresholds for SNV transmission, thereby linking community assembly to human disease risk. Although dietary overlap may partly reflect shared habitat associations among species, the stronger effect of diet relative to other traits suggests that resource competition plays a central role.

Recent frameworks emphasise additive versus substitutive assembly, sampling effects, community nestedness and directional diversity declines in shaping diversity‐disease relationships (Rohr et al. 2020). Our analysis provides powerful insights regarding how the interplay between these processes drives diversity disease relationships. While nestedness may influence disease patterns (Johnson et al. 2013), we found that nestedness, per se, did not drive our results. In fact, both communities were significantly “anti‐nested” in distinct ways (Table S18; Figures S6 and S7). We found that rodent communities were structured by environmental filtering and competitive exclusion, rather than by a consistent sequence of species loss or colonisation. Moreover, our findings demonstrate that the same type of assembly process—substitutive—can yield contrasting diversity–disease outcomes depending on which species are present and how they respond to changes in diversity. Only a subset of species acted as strong competitors, suggesting that sampling effects—where higher richness increases the likelihood of including an effective competitor—may have contributed to the dilution effect in some communities. Linking resource competition to dietary traits clarifies which species mediate community structure and disease outcomes, offering targets for management.

Although our sites experienced little anthropogenic disturbance, our results have implications for community disassembly, because local assembly rules set the stage for species loss. In dilution communities, disassembly would likely increase deer mouse density and infection risk, because community structure strongly favours the host, allowing it to rebound as diversity loss removes competitors. In contrast, in non‐dilution communities strong competitors keep deer mouse densities low and diversity loss is unlikely to increase disease risk unless those key competitors are lost first. Because deer mice have a broad environmental tolerance, disassembly favours deer mice except when environmental conditions advantage their competitors. Of course, directional disassembly could override these processes if, for example, anthropogenic disturbances specifically select for or against competent host species (e.g., Lyme disease or a plant fungal disease, Ostfeld and LoGiudice 2003; Liu et al. 2018). Either way, community assembly theory provides a framework for integrating the critical elements for mechanistically understanding how assembly and disassembly processes influence disease patterns.

A recent review suggests that host regulation drives diversity–disease relationships in rodent–hantavirus systems in the Americas: studies showing lower host density with higher diversity also showed reduced infection prevalence (Eleftheriou and Luis 2025). When diversity did not reduce host density, no clear disease pattern emerged. Our findings mirror this, supporting interspecific competition as a key mechanism linking diversity to host density and disease dynamics, modulated by environmental effects on community composition.

One limitation of our study is the lack of data on broader community diversity—particularly predators, which have been called a ‘neglected determinant’ of hantavirus dynamics (Guterres and de Lemos 2018). Predation can strongly influence host population and pathogen dynamics, as shown for Puumala hantavirus in bank voles in northern Europe (Khalil et al. 2016). While deer mice may be less vulnerable to predation than other rodents (Maron et al. 2010), predators likely help structure the overall rodent community and may indirectly affect deer mouse populations.

Spatial scale can influence diversity‐disease relationships (Cohen et al. 2016; Johnson et al. 2015; Rohr et al. 2020). Our study did not stratify across spatial scales but controlled for it: all 24 sites used the same sampling methods at the same scale. This approach demonstrates that local processes can generate contrasting diversity–disease patterns across the landscape. Focusing on the scale where organisms interact allowed us to identify mechanistic links to disease, while future work could test whether similar patterns emerge at larger or smaller scales.

Regarding temporal scale, we used long‐term averages of host density and disease risk across sites, which is appropriate for integrating community assembly because abiotic filtering effects on community composition operate over longer time scales. In contrast, predicting short‐term fluctuations in SNV infection within a site—such as changes following species turnover or arrival of competitors—requires mechanistic, dynamic models of host population and transmission processes (e.g., Luis et al. 2015 & 2018). Evaluating whether the patterns we identify here persist at finer temporal scales, and how they relate to within‐site temporal dynamics represents an important direction for future work.

By elucidating the community assembly processes determining infected host density, our results highlight a pathway from environmental filtering to human health risk—providing a mechanistic bridge between community ecology and zoonotic disease dynamics.

## Author Contributions

Both authors designed the study and wrote and edited the manuscript. A.D.L. performed all analyses.

## Funding

This work was supported by the National Science Foundation under Grant 2109828.

## Supporting information


**Figure S1:** Estimated daily capture probabilities, *pi*, for individuals of species 𝑖 (rows), given the individual was present, that is, was captured at least once in that primary session (month). Circles show the mean of the posterior distribution. Thick and thin lines show the 50% and 95% credible intervals, respectively. (For some species, e.g., *Peromyscus boylii* , the sample sizes were large enough that the 95% credible intervals fall within the plotted circle).
**Figure S2:** Phylogenetic relationships between rodents in the data from the Bininda‐Emonds et al. (2007) supertree.
**Figure S3:** Fit of the diet competition GLM for deer mouse abundance, the most supported of the trait models.
**Figure S4:** Nestedness matrix plot showing the presence (red) and absence (white) of rodent species across webs. Webs (rows) and species (columns; see Table S1 for species codes) have been reordered by the nestedness algorithm to highlight patterns of species co‐occurrence. In a perfectly nested matrix, presences would form a solid triangle in the upper left; deviations from this pattern contribute to the nestedness “temperature” statistic. The observed temperature indicates the degree of disorder, with lower values representing stronger nestedness. The observed temperature was 28.6, which was not significantly different from null expectations based on the quasiswap algorithm (*p* = 0.27), indicating no evidence of significant nestedness in the community structure.
**Figure S5:**. First 2 axes of the RDA showing sites coloured by community cluster. The RDA validates the clustering algorithm, showing that sites that cluster together have similar abiotic conditions, but the clustering approach allows us to assign rodent communities to distinct community types.
**Figure S6:** Nestedness matrix plot showing the presence (red) and absence (white) of rodent species across webs designated within the ‘dilution effect group’—those webs assigned to clusters 1, 2 and 4 by the clustering algorithm. Webs (rows) and species (columns; see Table S1 for species codes) have been reordered by the nestedness algorithm to highlight patterns of species co‐occurrence. The observed temperature was 23.8, which was not significantly different from null expectations based on the quasiswap algorithm (*p* = 0.27), indicating no evidence of significant nestedness in the community structure.
**Figure S7:** Nestedness matrix plot showing the presence (red) and absence (white) of rodent species across webs designated within the ‘non‐dilution effect group’—those webs assigned to clusters 3 and 5 by the clustering algorithm. Webs (rows) and species (columns; see Table S1 for species codes) have been reordered by the nestedness algorithm to highlight patterns of species co‐occurrence. The observed temperature was 25.5, which was not significantly different from null expectations based on the quasiswap algorithm (*p* = 0.45), indicating no evidence of significant nestedness in the community structure.
**Figure S8:** Equivalent to Figure 5 in main text, here showing infected host density rather than infection prevalence. (A) (Top) dendrogram of hierarchical clustering by environmental variables. Dendrogram tips are coloured by assigned environmental cluster. Beneath the dendrogram, shading shows relative values of environmental variables (rows) by site (columns). Rows indicated with the red bar (density of SNV‐infected deer mice, rodent diversity and deer mouse density) were not included in the clustering algorithm but are shown for comparison. To better understand what mediates a dilution effect, as a post hoc analysis, we grouped clusters 1, 2 and 4 together because they displayed a dilution effect (solid line, B) and groups 3 and 5 which did not (dashed line, B) and further analysed community relationships. (B) Relationship between mean rodent diversity by Simpson's D and mean SNV‐infected deer muse density (note log scale of *y*‐axis), (C) deer mouse density and SNV‐infected deer muse density and (D) rodent diversity and deer mouse density, (E) diversity on potential diet competition (the sum of competitor densities times their percent diet overlap with deer mice).
**Figure S9:** QQ plots and Diagnostics for all GLMs. Uniformity of simulated residuals was assessed using DHARMa; QQ plots showed no systematic deviations from model assumptions.
**Table S1:** Species 2‐letter abbreviations used in figures.
**Table S2:** Results from binomial GLM modelling SNV prevalence as a function of rodent diversity in figure 2A. Models included an indicator variable (“in Luis et al. 2018”) identifying webs previously analysed in Luis et al. (2018), allowing direct comparison between the original nine webs and the additional webs included in the expanded dataset. The variable and interaction were important, suggesting a different diversity‐disease relationship between those webs included vs. not included in the previous study. See Figure S9a for diagnostic plot.
**Table S3:** Results from Tweedie GLM modelling infected host density as a function of rodent diversity in Figure 2B. Models included an indicator variable (“in Luis et al. 2018”) identifying webs previously analysed in Luis et al. (2018), allowing direct comparison between the original nine webs and the additional webs included in the expanded dataset. The variable and interaction were important, suggesting a different diversity‐disease relationship between those webs included vs. not included in the previous study. See Figure S9b for diagnostic plot.
**Table S4:** Summary of Redundancy Analysis results including eigenvalues and explanatory power of constrained axes (comprised of environmental variables) labelled as ‘RDA’ and unconstrained axes labelled as ‘PC’ (additional correlations between species abundances).
**Table S5:** Significance of permutation tests on predictor variables from the RDA, with 1000 permutations.
**Table S6:** Significance of permutation tests on the canonical axes from the RDA, with 1000 permutations.
**Table S7:** Model comparisons of biotic regressions with web as the replicate. AIC weight 1 shows the AIC weights when considering only the models with traits. AIC weight 2 shows weights when adding productivity to the best trait model.
**Table S8:** Results of best GLM for deer mouse abundance (web as replicate) using functional traits. See Figure S9c for diagnostic plot.
**Table S9:** Model comparisons of biotic regressions with site as the replicate, using means of webs per site. AIC weight 1 shows the AIC weights when considering only the models with traits. AIC weight 2 shows weights when adding productivity to the best trait model.
**Table S10:** Results of best GLM for deer mouse abundance (site as replicate) using functional traits. See Figure S9d for diagnostic plot.
**Table S11:** Summary of nestedness and co‐occurrence metrics based on rodent presence–absence data. The table reports the observed value of each metric (Statistic), the mean of the null distribution (from 999 simulations using the “quasiswap” algorithm), and the standardised effect size (SES). *p*‐value is two‐sided, where the alternative hypothesis is significantly nested. Matrix temperature reflects deviation from perfect nestedness (lower values indicate stronger nestedness). *C*‐score = co‐occurrence metric based on checkerboard units; lower values than null model indicate anti‐nestedness. NODF = Nestedness metric based on Overlap and Decreasing Fill; lower values than null indicate anti‐nestedness.
**Table S12:** Standardised estimates, standard errors (SE) and *p*‐values for the paths in the structural equation model (SEM) presented in the main text (Figure 4). AIC = 199.39.
**Table S13:** Standardised estimates, standard errors (SE) and *p*‐values for the paths in the SEM including a direct effect of abiotic variables on deer mouse abundance. There is not support for adding the additional path. AIC = 201.39.
**Table S14:** Standardised estimates, standard errors (SE) and *p*‐values for the paths in the SEM including a link from rodent diversity to deer mouse abundance. There is not strong support for the additional path. AIC = 200.11.
**Table S15:** Binomial GLM model comparisons for Figure 5B, examining how Simpson's diversity index and grouping of clusters affects SNV prevalence. There was support for grouping the blue community cluster 4 with clusters 1 and 2 as part of the ‘Dilution group’. There was also support for the additive (+) model over the model with an interaction (*) term.
**Table S16:** Results of the binomial GLM for how rodent diversity affects SNV prevalence, with sites grouped by dilution effect or non‐dilution effect clusters, represented in Figure 5B. See Figure S9e for diagnostic plot.
**Table S16:** Results of the binomial GLM for how rodent diversity affects SNV prevalence, with sites grouped by dilution effect or non‐dilution effect clusters, represented in Figure 5B. See Figure S9e for diagnostic plot.
**Table S17:** Partitioning of beta diversity into turnover and nestedness components based on Sorensen dissimilarity. Total dissimilarity (*βSOR*) was divided into the turnover component (*βSIM*), representing species replacement among sites, and the nestedness component (*βSNE*) representing species loss or gain without replacement. Results indicate that most variation in community composition was driven by turnover rather than nestedness.
**Table S18:**. Summary of nestedness and co‐occurrence metrics based on rodent presence–absence data for the ‘Dilution effect group’ and ‘Non‐dilution effect group’. The table reports the observed value of each metric (Statistic), the mean of the null distribution (from 999 simulations using the “quasiswap” algorithm), and the standardised effect size (SES). *p*‐value is two‐sided, where the alternative hypothesis is significantly nested. Matrix temperature reflects deviation from perfect nestedness (lower values indicate stronger nestedness). C‐score = co‐occurrence metric based on checkerboard units; lower values than null model indicate anti‐nestedness. NODF = Nestedness metric based on Overlap and Decreasing Fill; lower values than null indicate anti‐nestedness.
**Table S19:** Results of the binomial GLM for how deer mouse density affects SNV prevalence, with sites grouped by dilution effect or non‐dilution effect, represented in Figure 5C. See Figure S9f for diagnostic plot.
**Table S20:** Results of the Tweedie GLM for how rodent diversity affects deer mouse density, with sites grouped by dilution effect or non‐dilution effect, represented in Figure 5D. See Figure S9g for diagnostic plot.
**Table S21:** Results of the Tweedie GLM for how rodent diversity affects diet competition, with sites grouped by dilution effect or non‐dilution effect, represented in Figure 5E. Diet competition was calculated as the sum of competitor densities times their similarity in diet overlap with deer mice. See Figure S9h for diagnostic plot.

## Data Availability

All data and code used in this study are available in Zenodo at https://doi.org/10.5281/zenodo.18744824.
