# Supplementary material for: Community Assembly Reveals How Environmental Controls Over Rodent Competition Drive Deer Mouse Density and Hantavirus Infection
Source: Ecol Lett. 2026 Mar 30;29(4):e70374. doi: 10.1111/ele.70374 (PMC13035436; doi:10.1111/ele.70374)
Supplement: Supplementary file 1 — Figure S1: Estimated daily capture probabilities, pi, for individuals of species 𝑖 (rows), given the individual was present, that is, was captured at least once in that primary session (month). Circles show the mean of the posterior distribution. Thick and thin lines show the 50% and 95% credible intervals, respectively. (For some species, e.g., Peromyscus boylii , the sample sizes were large enough that the 95% credible intervals fall within the plotted circle). Figure S2: Phylogenetic relationships between rodents in the data from the Bininda‐Emonds et al. (2007) supertree. Figure S3: Fit of the diet competition GLM for deer mouse abundance, the most supported of the trait models. Figure S4: Nestedness matrix plot showing the presence (red) and absence (white) of rodent species across webs. Webs (rows) and species (columns; see Table S1 for species codes) have been reordered by the nestedness algorithm to highlight patterns of species co‐occurrence. In a perfectly nested matrix, presences would form a solid triangle in the upper left; deviations from this pattern contribute to the nestedness “temperature” statistic. The observed temperature indicates the degree of disorder, with lower values representing stronger nestedness. The observed temperature was 28.6, which was not significantly different from null expectations based on the quasiswap algorithm (p = 0.27), indicating no evidence of significant nestedness in the community structure. Figure S5:. First 2 axes of the RDA showing sites coloured by community cluster. The RDA validates the clustering algorithm, showing that sites that cluster together have similar abiotic conditions, but the clustering approach allows us to assign rodent communities to distinct community types. Figure S6: Nestedness matrix plot showing the presence (red) and absence (white) of rodent species across webs designated within the ‘dilution effect group’—those webs assigned to clusters 1, 2 and 4 by the clustering algorithm. Webs (rows) [file ELE-29-0-s001.pdf]

Supporting Information Appendix

for Luis & Pearson. 2026. Community assembly reveals how environmental controls over rodent competition drive deer mouse density and hantavirus infection. *Ecology Letters*.

SI Methods

Rodent Data

Each of the 24 rodent trapping webs contained 148 Sherman traps spread over 3.14 ha including 12 100-m transects radiating from a central point. Sites in Arizona and New Mexico were trapped monthly and in Colorado every 6 weeks, as weather allowed (see Mills et al. (1999) for details). All sites were consistently trapped from May to September, but a third of the sites had few winter trapping events. Therefore, we restricted the rodent dataset to the months of May to September. This is further justified by the overlap of this seasonal interval with the spring and summer breeding season and time when plant and invertebrate food resources are most abundant. It also corresponds to the timing of most human SNV spillover events, thereby linking our results with the period of greatest human risk (Whitmer *et al.* 2024). To capture broadscale patterns reflecting community assembly processes (Keddy & Laughlin 2021), rather than within-site temporal dynamics, we calculated mean species abundances for all rodent species, mean deer mouse infection prevalence, and mean density of infected deer mice per web during the growing season (from May to September) over the 12-year study period, adjusting for species-specific capture rates (next section).

Generally, for a monthly trapping event, trapping occurred for 3 consecutive nights. Traps were set in the evenings, baited with peanut butter and oats, cracked corn or mixed grain. Cotton or polyester bedding was added for nesting material and insulation. Traps were checked in the mornings, and animals were anesthetized, ear-tagged, weighed, and a blood sample was taken to test for antibodies against SNV at the CDC, Atlanta. Because SNV is a chronic infection that is not cleared, antibodies are an appropriate indicator of current infection (Bagamian *et al.* 2013).

Estimating Abundance

We first estimated species abundances over time at each web by estimating a species-specific capture probability and using that to adjust number of captures per primary occasion, according to $N_{i,t} = C_{i,t}/(1-(1-p_i)^{d_t})$, where $N_{i,t}$ and $C_{i,t}$ are the estimate of abundance and number of unique individuals captured, respectively, for species i, at primary occasion (month), t. p_i is the estimated capture probability for individuals of species i, given the individual was present, i.e, was captured at least once in that primary session (month). d_t is the number of secondary occasions per primary occasion (t) i.e., number of consecutive days trapped that month. p_i was estimated using Bayesian capture-mark-recapture models (Kéry & Schaub 2012) implemented in the *R2jags* package (Su & Masanao Yajima 2021) and is presented in Fig. S1.

Environmental Data

We obtained vegetation cover and productivity estimates for the CDC trapping webs using the Rangeland Analysis Platform, which provides data at a 30m-by-30m spatial resolution. We created shape files using the web coordinates as the central point and with a 3.14ha area to

encompass each web. The vegetation cover dataset partitions ground cover into percent cover of annual forbs and grasses, perennial forbs and grasses, shrubs, trees, litter, and bare ground at the annual scale (Allred *et al.* 2021). The rangeland production dataset reports estimates of total new herbaceous above ground biomass (annual and perennial forbs and grasses in lbs/acre) over each 16-day period (hereby referred to as productivity) which is then summed to an annual total (Jones *et al.* 2021). We used the mean of the annual estimates from 1994-2006.

Herbaceous above ground biomass was selected to represent productivity because it captures the herbaceous resources most relevant to small mammals and deer mice for this region. Long-term trend analyses show strong population responses of deer mice to precipitation-driven increases in herbaceous plant productivity linked to increased food resources (seeds and invertebrates) in the southwestern US (Carver *et al.* 2015; Yates *et al.* 2002). The RAP data provide the best metric for herbaceous plant productivity. In contrast, NDVI, while commonly used, correlates more strongly with tree cover in our study region and therefore is less indicative of deer mouse resource availability.

We obtained climatological summaries from Daymet (Thornton *et al.* 2022) at a 1km spatial resolution using the *daymetr* package (Hufkens *et al.* 2018), including daily total precipitation in mm/day (sum of all forms converted to water), daily temperature minima and maxima (°C), and snow-water equivalent (km/m^2) – a measurement of the amount of water contained within the snowpack. To obtain monthly variables, we summed total precipitation for the month, and took the average daily minimum temperature and average daily maximum temperature. We reported the maximum amount of snow-water equivalent (referred to as snow) for each month. For analyses, we used the mean of the monthly estimates for all months from 1994-2006.

For all analyses, we standardized the environmental variables to have a mean of 0 and variance of 1 using the *decostand* function in the *vegan* R package (Oksanen *et al.* 2022).

Phylogenetic and Functional Trait Data

Using the phylogenetic supertree from Bininda-Emonds et al. (2007), we calculated the phylogenetic distance between deer mice and each of the other rodent species in the dataset, using the *cophenetic.phylo* function from the *ape* package (Paradis & Schliep 2019). We obtained data on diet and activity (nocturnal, diurnal, crepuscular) at the species level from the EltonTraits 1.0 database (Wilman *et al.* 2014), in which diet was partitioned into percent of diet belonging to different categories (e.g., fruit, nectar, seed, plant, invertebrates, scavenge, etc.). We calculated the sum of differences between deer mice and each of the rodent species for each category. For example, montane voles, *Microtus montanus*, were reported to have a diet consisting of 80% plants and 20% seeds. Deer mice were reported to have a diet consisting of 50% invertebrates, 20% fruit, 20% seeds, and 10% scavenge. The absolute value of differences for each of these food categories was calculated as: plants = 80 (voles) – 0 (mice) = 80; seeds = 20 – 20 = 0; invertebrates = 50; fruit = 20, scavenge = 10. Then we summed those differences to get a total diet difference between montane voles and deer mice of 160. For activity, we used the EltonTraits database to classify each species as nocturnal or not. We obtained average adult body mass for each species from the amniote life-history database (Myhrvold *et al.* 2015). We calculated the difference in logged mass (for normality) between each species and deer mice. The above data were distances from deer mice. We used these distances to calculate similarity, standardized between 0 and 1, where similarity = 1 – distance/(max distance) for each variable.

Therefore, higher values of similarity represent more niche overlap— a potential proxy for strength of competition, proportional to a Lotka-Volterra competition coefficient.

Generalized Linear Models

For all analyses where SNV prevalence was the response variable, we used a binomial GLM with a logit link and weights equal to the number of sampling occasions per web.

For all analyses where density/abundance was the response variable (deer mouse abundance or infected host density), we used a Tweedie distribution fit via the *glmmTMB* function (McGillycuddy *et al.* 2025). The Tweedie error distribution is appropriate for continuous (because of averaging), non-negative response variables that include a mixture of zeros and positive values. The Tweedie distribution is a compound Poisson–Gamma distribution in which the variance scales with the mean as $\mathrm{Var}(Y) = \varphi\,\mu^p$, where p (the variance power parameter) determines the mean–variance relationship and the probability of observing zeros. Unlike zero-inflated models, zeros arise naturally in the Tweedie distribution through the Poisson component of the compound Poisson–Gamma process. The variance power parameter (p) was estimated by profile likelihood using the *tweedie.profile* function, evaluating values of p from 1.1 to 1.9. The value maximizing the profile likelihood ($p = 1.83$ for deer mouse abundance) was then fixed and used consistently across all candidate models to ensure comparability of model fits and information-theoretic model selection.

We used simulation-based diagnostics implemented in the DHARMa package (Hartig 2024). This approach generates residuals by simulating responses from the fitted model and comparing observed values to the simulated distribution. We evaluated uniformity of residuals using quantile–quantile plots and formal tests, assessed dispersion and zero inflation, and

inspected residuals versus predicted values for systematic patterns. These diagnostics are all plotted in Figure S9 and indicated that the fitted models adequately captured the mean–variance structure of the data.

For all figures, confidence intervals for model predictions were obtained using a parametric bootstrap. We repeatedly simulated new response data from the fitted GLM, refit the model to each simulated dataset, and generated predicted values for a fixed set of covariate values. This procedure was repeated 1,000 times. For each prediction, the median of the bootstrap distribution was used as the fitted value, and 95% confidence intervals were calculated from the 2.5th and 97.5th percentiles of the bootstrap predictions. This approach propagates both parameter uncertainty and sampling variability into the estimated confidence intervals.

Biotic emphasis for evaluating dilution effects

We first analyzed the dataset using the traditional biotic approach, in which we examined the relationship between average rodent diversity and average SNV prevalence or average density of SNV-infected deer mice among webs without regard to community composition and underlying structuring processes. Although evenness would be the appropriate diversity metric for the encounter reduction mechanism, for the host regulation mechanism, a diversity metric that also accounts for species richness in addition to relative abundance would be more appropriate. Therefore, for diversity, we calculated inverse Simpson's diversity index, $D = 1/\sum_{i=1}^{R} s_i^2$, for each web per month, where s_i is the proportional abundance of species i, and R is the rodent richness (total number of rodent species trapped at that web that month). We examined the average SNV risk as a function of average D over the growing season. We modeled web-level mean prevalence using a binomial GLM with a logit link and weights equal to the number of

sampling occasions per web. We also modeled web-level mean density of infected deer mice using a Tweedie distribution fit via the *glmmTMB* function.

Biotic effects on host abundance and linkages to traits

We evaluated how biotic interactions influenced deer mouse (*pm*) abundance, considering species traits – phylogenetic relatedness, body size, nocturnality, diet overlap (H_2, H_3). Abundance was modeled using Tweedie GLMs to accommodate non-negative continuous data with zeros, with the variance power parameter estimated by profile likelihood (p = 1.83) and held constant across models for valid AIC comparison. See SI Appendix for more details.

Each regression took the form, $pm_j = \beta_0 + \beta_1 \cdot \sum_i \alpha_i N_{i,j}$, where, $N_{i,j}$ is the abundance of species i (excluding deer mice) at web j, and α_i is similarity between species i and deer mice, based on each trait. We ran four independent models, each with a different α: 1) phylogenetic similarity, 2) dietary overlap, 3) body mass similarity, and 4) nocturnality (1 for nocturnal species, 0 otherwise). Each model estimated two parameters, β_0 (intercept) and β_1 (slope). Here, $\alpha_i \beta_1$ is equivalent to a Lotka-Volterra competition coefficient for species i (based solely on the examined similarity in phylogeny or trait). Therefore, $\sum_i \alpha_i N_{i,j}$ represents the total competitive pressure on deer mice over all rodent species based on niche overlap of that trait. For comparison, we also tested a model using Simpson's diversity index as the predictor. We compared the five models by AIC.

Because each of the individual trait regressions includes the sum of rodent species abundances (besides deer mice) weighted by similarity in the focal trait, although the weightings are different between models, the variables are highly correlated (R~0.75), which creates strong

multicollinearity if multiple traits were included in a single model. Including them together would therefore confound parameter estimates and reduce interpretability. Therefore, we tested each trait separately to determine which best predicts deer mouse responses. This approach allows us to identify the dominant biotic mechanism linked to deer mouse responses while avoiding statistical artifacts due to correlated predictors.

Lotka-Volterra theory also indicates that competition is influenced by the environmental carrying capacity, *K*, specifically that equilibrium abundance at web j should equal $K_j - \sum_i \alpha_i N_{i,j}$ (Gotelli 1998). Therefore, we assessed whether including a proxy for *K* explained more variation than the best competition model. If we assume carrying capacity is a linear function of productivity, $K_j = \beta_0 + \beta_1 \cdot productivity_j$, then we can model deer mouse abundance with the linear regression, $pm_j = \beta_0 + \beta_1 \cdot productivity_j + \beta_2 \cdot \sum_i \alpha_i N_{i,j}$ and determine if this model has a lower AIC than the best competition model without productivity.

We used web as the replicate because there were some differences in rodent community composition and environmental variables across webs within a site. Our inference focuses on explaining broad-scale variation among sites; including site as a random effect would instead emphasize within-site contrasts and absorb the between-site variation of interest. To assess potential pseudoreplication, we repeated all regressions at the site level, averaging across webs, and report those results in the SI Appendix.

Structural Equation Models

Because the hierarchical assembly processes explored required various *ad hoc* analyses, we fit structural equation models (SEMs) to explore the interactions among key driver and response

metrics - abiotic factors, competitor communities, deer mouse abundance, and SNV risk. The models were fit using the lavaan package (Rosseel 2012). SEM focuses on causal pathways rather than response distributions. Estimation and model fit rely on covariance structures that assume approximately linear and homoscedastic relationships. To better meet these assumptions for bounded data containing zeroes, prevalence and density measures were transformed prior to analysis. This approach improves statistical inference while preserving the underlying causal structure of the model. Distributionally explicit GLMs (binomial and Tweedie) were fitted separately for primary inference on prevalence and density.

Because prevalence values were averaged across sampling months and therefore do not correspond to binomial trials, prevalence was treated as a continuous proportion. To stabilize variance and accommodate true zero values, we applied a logit transformation with a small, data-derived offset. Specifically, prevalence p was transformed as $\log((p+\varepsilon)/(1-p+\varepsilon))$, where ε was set to the minimum of the non-zero prevalence values (Warton & Hui 2011). Results were not sensitive to choice of ε across a plausible range.

For densities (N; deer mouse density, competitor densities that were a part of the competition term, as well as infected host density), we used the $\log(N+c)$ transformation, where c was set to the first quartile of the non-zero values for all species abundances. This data-derived offset aims to reduce sensitivity to zeroes and extreme skew (Stahel 2002). Conclusions were not sensitive to c over the range 0.004 – 1. These transformations were used solely as working scales for SEM, while distributionally explicit generalized linear models were used for primary inference.

Abiotic variables (temperature, precipitation, snow water equivalent, and elevation) were summarized using principal components analysis (PCA), and the first three components were

summed into a single abiotic index based on loadings. This treats the retained axes as equally weighted contributors to a composite index – a simplifying assumption, given the low sample size for an SEM. For biotic interactions, we included the covariate(s) that had the most support from the biotic regressions (above). All variables were standardized (mean = 0, SD = 1).

We evaluated competing causal hypotheses using a set of a priori SEMs that differed in the inclusion and direction of biologically motivated pathways. In particular, we tested whether abiotic conditions primarily structured competitor communities rather than directly affecting deer mouse abundance, and whether diversity metrics (Simpson's index) explained additional variation. Models were fit using maximum likelihood estimation and compared using Akaike's Information Criterion (AIC). Model fit was additionally assessed using standard SEM fit indices (CFI, RMSEA, SRMR) to identify gross misspecification.

Cluster Analysis

We applied cluster analyses as a *post hoc* tool for visualizing how environmental filtering shapes community structure and mediates diversity-disease relationships. This approach allowed us to assign rodent communities to distinct community types based on environmental factors, thereby mirroring the results from the RDA but overcoming the RDA constraint to continuous output. First, we calculated the Euclidean distance between each web based on environmental conditions to create a dissimilarity matrix, using the *dist* function on the standardized environmental variable matrix. We compared several hierarchical clustering algorithms, including linkage agglomerative clustering using chord distances, complete-linkage agglomerative clustering, unweighted pair-group method using averages (UPGMA) agglomerative clustering, and centroid clustering, using the *hclust* function. See Borcard et al. (2018) for more information. We tested

which algorithm performed best by cophenetic correlation, by first calculating the cophenetic distance matrix using the cophenetic function, and then calculating the correlation to the environmental dissimilarity matrix.

We evaluated cluster number using two independent optimization approaches. First, we calculated average silhouette width (Rousseeuw 1987) for solutions ranging from $k = 2$ to $k = n - 1$, using the silhouette function applied to the hierarchical clustering solution. The silhouette criterion, which measures within-cluster cohesion relative to between-cluster separation, identified an optimum at $k = 4$. Second, we evaluated the matrix correlation approach of Borcard et al. (2018), which assesses how well a given clustering reproduces the original environmental distance matrix. For each candidate k, we computed a group-distance matrix and calculated its Pearson correlation with the original Euclidean distance matrix. This criterion identified an optimum at $k = 7$.

Because these optimization criteria emphasize different aspects of cluster structure and yielded different optima, we selected $k = 5$ as an intermediate solution that balanced statistical support with biological interpretability. Solutions with $k \leq 4$ merged environmentally distinct sites, whereas solutions with $k \geq 6$ produced several clusters containing only 1–2 webs, limiting their usefulness for interpreting community–disease relationships. The $k = 5$ solution captured major environmental gradients while retaining sufficient replication within clusters to support post hoc analyses.

SI Results

Structural Equation Models

In the main text, we present results using SNV prevalence in deer mice as the disease metric. Alternatively, using density of SNV-infected deer mice as the disease metric yields the same conclusions. Deer mouse abundance had a strong positive effect on the density of infected hosts ($\beta = 0.88$), and additional paths involving rodent diversity (Simpson's index) were still not supported. The SEM demonstrated adequate fit to the data ($\chi^2 = 11.84$, df = 4, p = 0.019, CFI = 0.92, SRMR = 0.07). The model explained 78% in infected host density.

SI Discussion

For SNV, prevalence and infected host density were strongly correlated with deer mouse density above ~3 mice/ha, consistent with density dependent transmission (Luis *et al.* 2015). Within a Montana site, sustained densities above 17 mice/ha were needed for transmission (Luis *et al.* 2015). While these thresholds differ, they reflect different scales: the lower threshold emerges from spatially averaged data across sites, whereas the higher threshold reflects local, within-site dynamics over time. Short-lived spikes above 17 mice/ha can enable transmission even if the average remains low due to high temporal variability. Additionally, the transmission rates estimated in Luis *et al.* (2018) for some of these sites were significantly higher than for the Montana site, which would decrease the threshold (critical host density). Thus, our results

demonstrate that host density—and consequently transmission risk—is influenced by broader community and environmental processes, while not contradicting within-site density requirements for transmission.

SI Figures

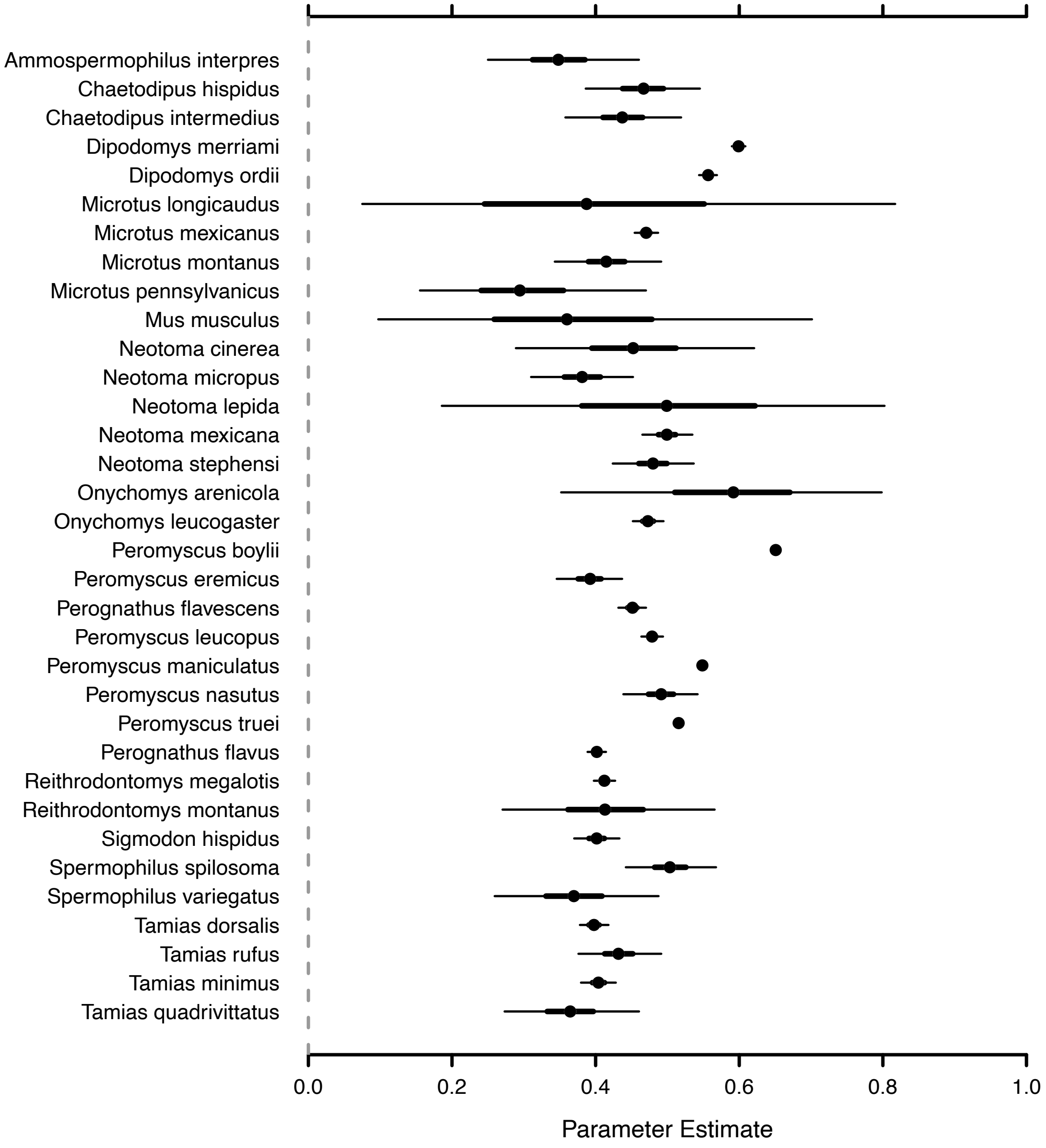

Figure S1. Estimated daily capture probabilities, p_i, for individuals of species i (rows), given the individual was present, i.e, was captured at least once in that primary session (month). Circles show the mean of the posterior distribution. Thick and thin lines show the 50% and 95% credible intervals, respectively. [For some species, e.g., *Peromyscus boylii*, the sample sizes were large enough that the 95% credible intervals fall within the plotted circle.]

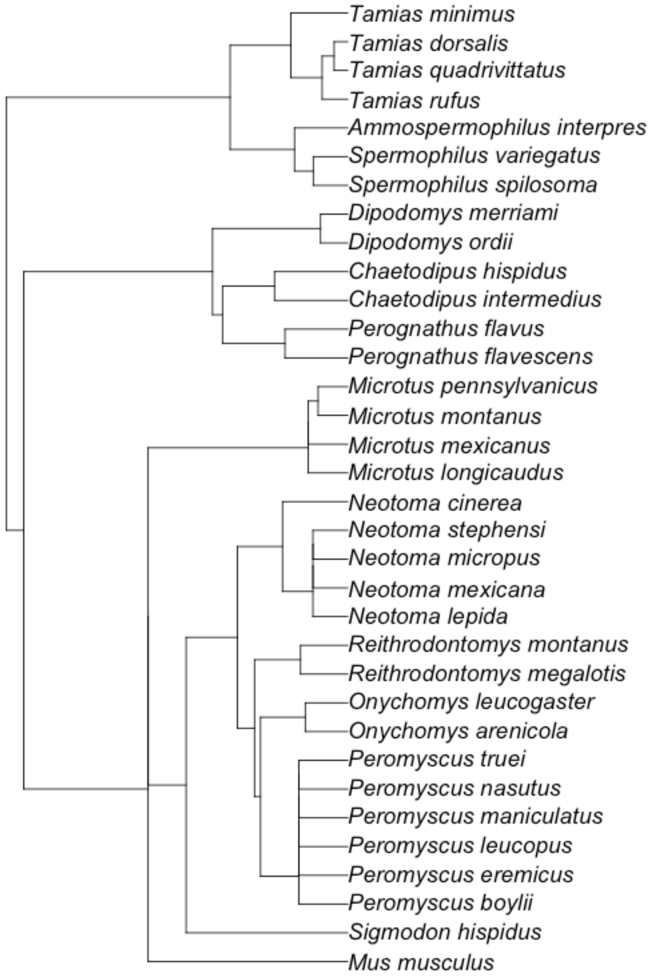

Figure S2. Phylogenetic relationships between rodents in the data from the Bininda-Emonds *et al.* (2007) supertree.

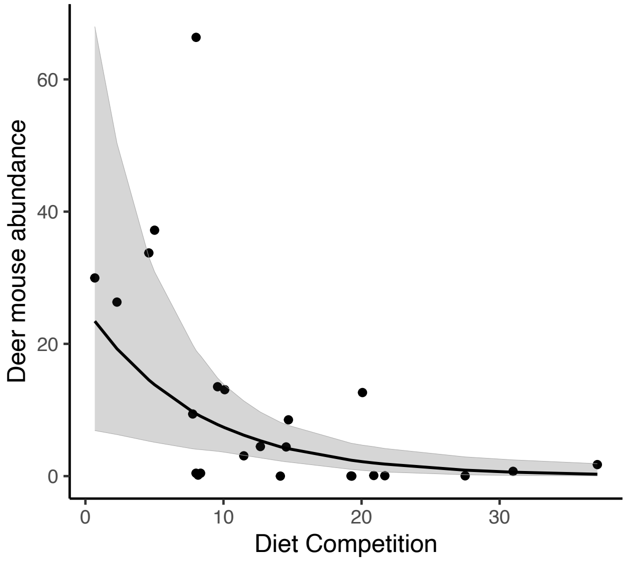

Figure S3. Fit of the diet competition GLM for deer mouse abundance, the most supported of the trait models.

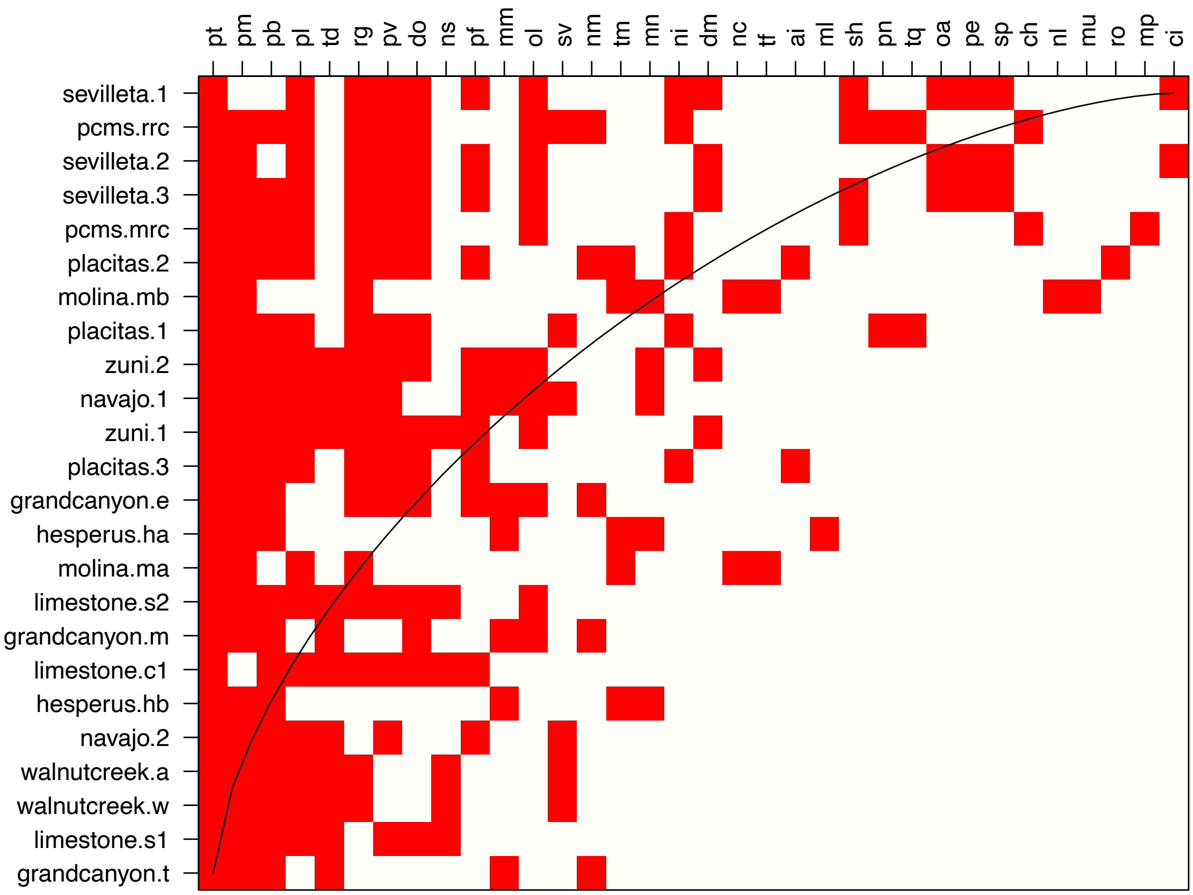

Figure S4. Nestedness matrix plot showing the presence (red) and absence (white) of rodent species across webs. Webs (rows) and species (columns; see Table S1 for species codes) have been reordered by the nestedness algorithm to highlight patterns of species co-occurrence. In a perfectly nested matrix, presences would form a solid triangle in the upper left; deviations from this pattern contribute to the nestedness "temperature" statistic. The observed temperature indicates the degree of disorder, with lower values representing stronger nestedness. The observed temperature was 28.6, which was not significantly different from null expectations based on the quasiswap algorithm (p = 0.27), indicating no evidence of significant nestedness in the community structure.

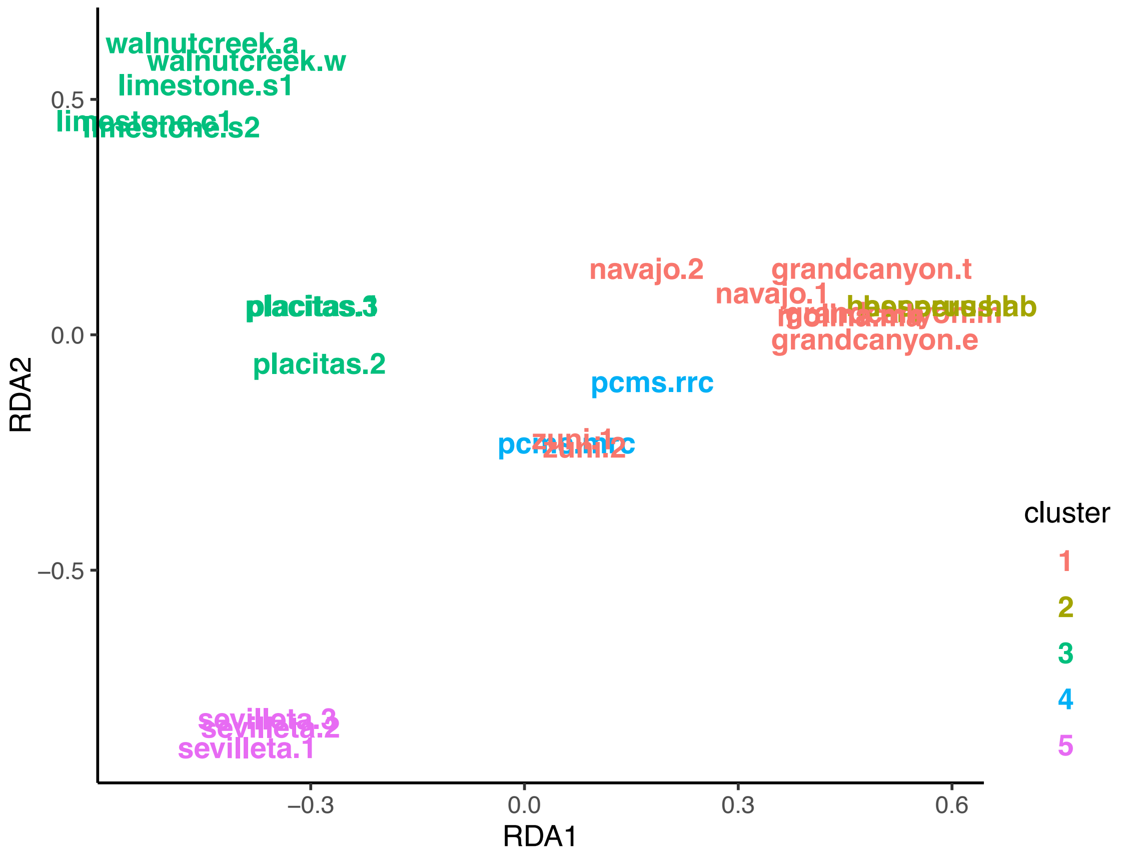

Figure S5. First 2 axes of the RDA showing sites colored by community cluster. The RDA validates the clustering algorithm, showing that sites that cluster together have similar abiotic conditions, but the clustering approach allows us to assign rodent communities to distinct community types.

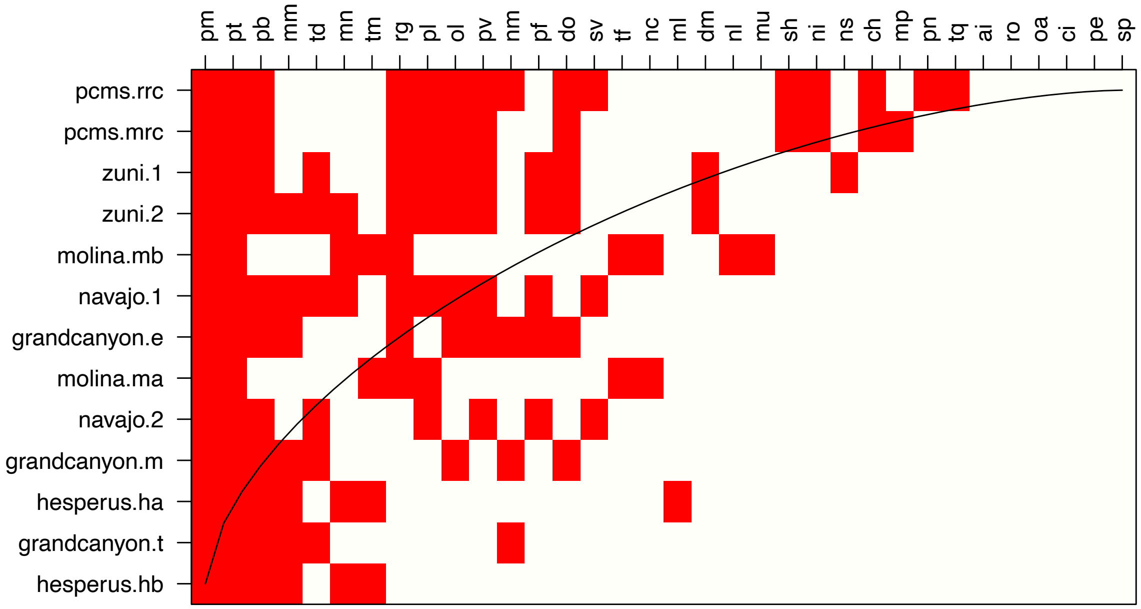

Figure S6. Nestedness matrix plot showing the presence (red) and absence (white) of rodent species across webs designated within the 'dilution effect group' – those webs assigned to clusters 1, 2, and 4 by the clustering algorithm. Webs (rows) and species (columns; see Table S1 for species codes) have been reordered by the nestedness algorithm to highlight patterns of species co-occurrence. The observed temperature was 23.8, which was not significantly different from null expectations based on the quasiswap algorithm (p = 0.27), indicating no evidence of significant nestedness in the community structure.

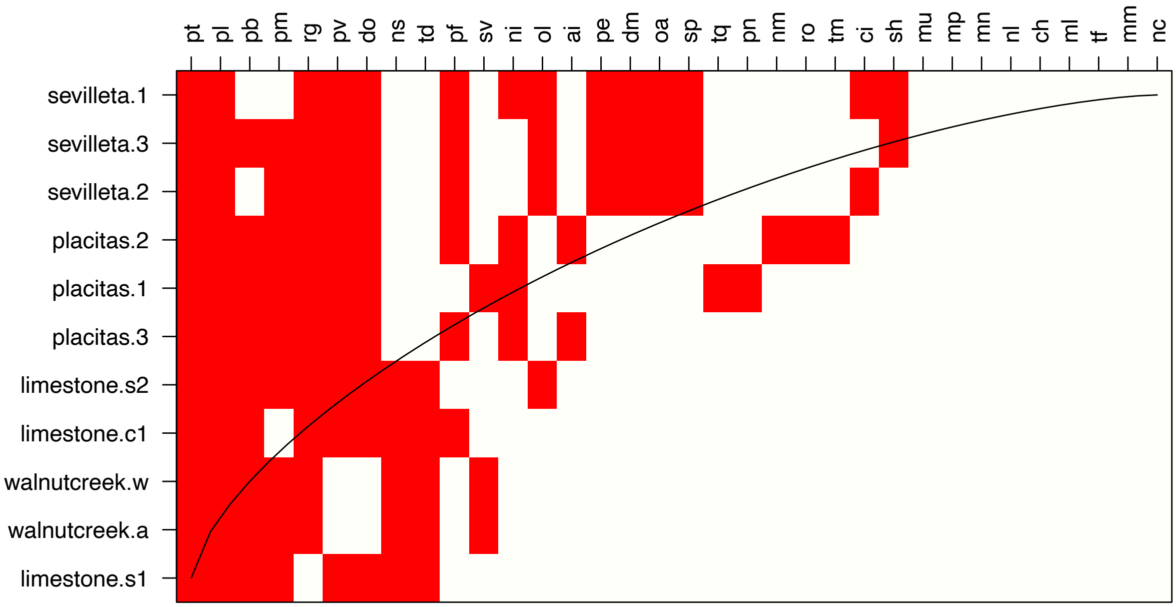

Figure S7. Nestedness matrix plot showing the presence (red) and absence (white) of rodent species across webs designated within the 'non-dilution effect group' – those webs assigned to clusters 3 and 5 by the clustering algorithm. Webs (rows) and species (columns; see Table S1 for species codes) have been reordered by the nestedness algorithm to highlight patterns of species co-occurrence. The observed temperature was 25.5, which was not significantly different from null expectations based on the quasiswap algorithm (p = 0.45), indicating no evidence of significant nestedness in the community structure.

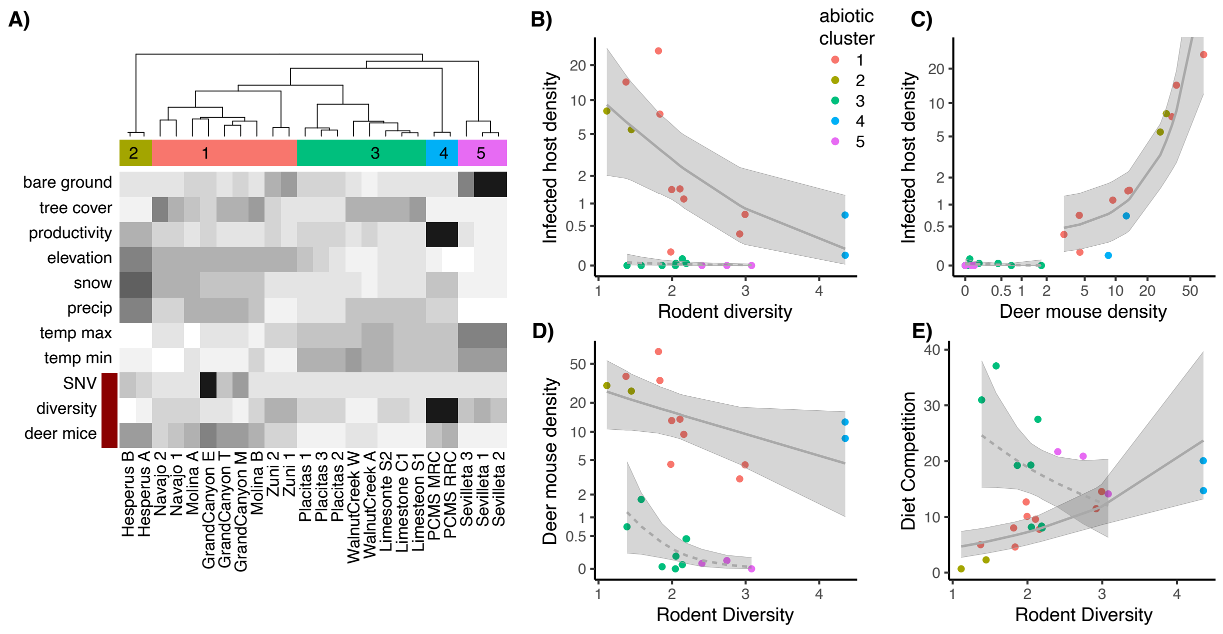

Figure S8. Equivalent to figure 5 in main text, here showing infected host density rather than infection prevalence. (**A**) (Top) dendrogram of hierarchical clustering by environmental variables. Dendrogram tips are colored by assigned environmental cluster. Beneath the dendrogram, shading shows relative values of environmental variables (rows) by site (columns). Rows indicated with the red bar (density of SNV-infected deer mice, rodent diversity, and deer mouse density) were not included in the clustering algorithm but are shown for comparison. To better understand what mediates a dilution effect, as a post hoc analysis, we grouped clusters 1, 2, and 4 together because they displayed a dilution effect (solid line, B) and groups 3 and 5 which did not (dashed line, B) and further analyzed community relationships. (**B**) Relationship between mean rodent diversity by Simpson's D and mean SNV-infected deer muse density (note log scale of y-axis), (**C**) deer mouse density and SNV-infected deer muse density, and (**D**) rodent diversity and deer mouse density, (**E**) diversity on potential diet competition (the sum of competitor densities times their percent diet overlap with deer mice).

Figure S9. QQ plots and Diagnostics for all GLMs. Uniformity of simulated residuals was assessed using DHARMa; QQ plots showed no systematic deviations from model assumptions.

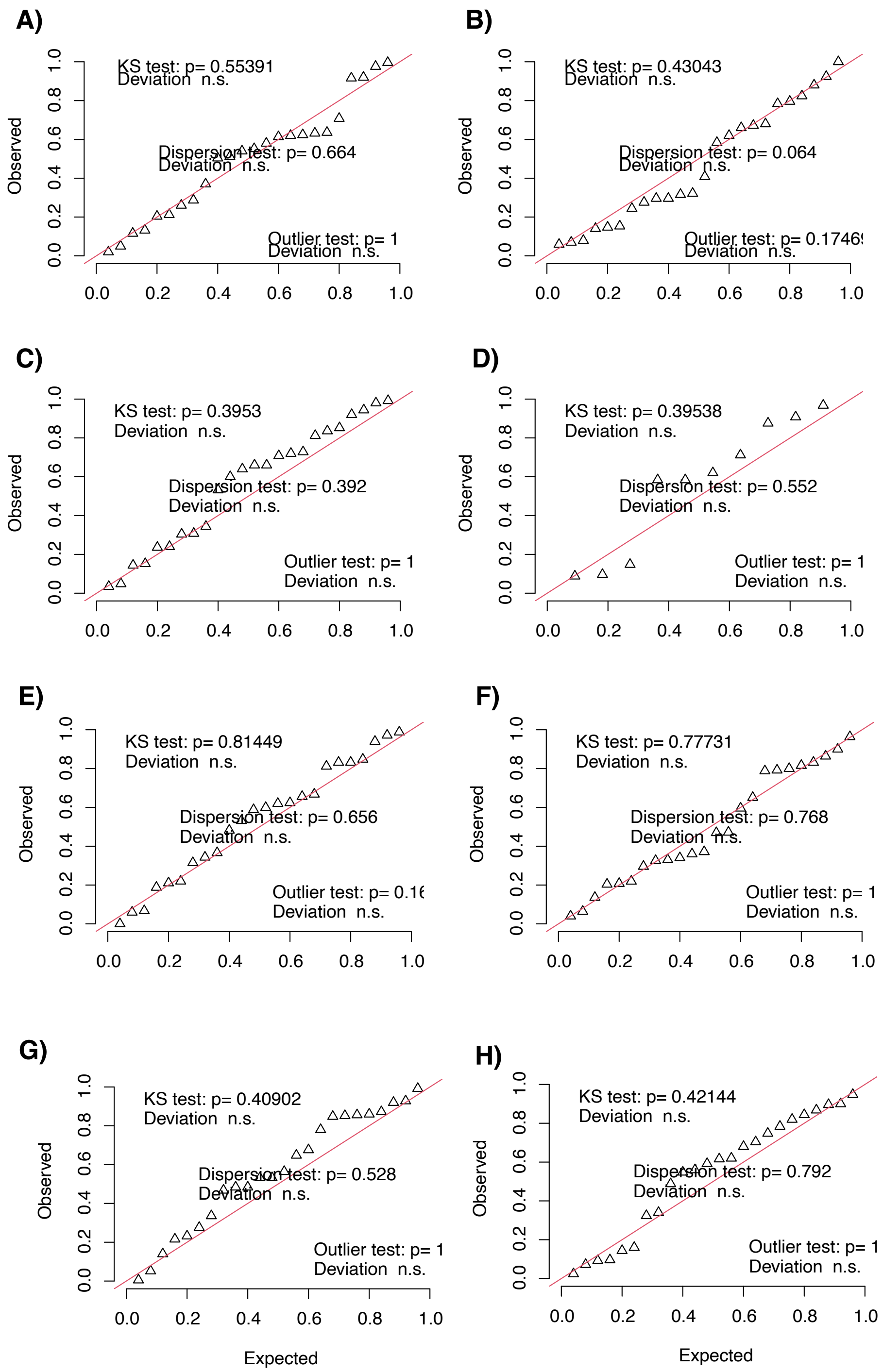

A) Binomial GLM in Figure 2A.

B) Tweedie GLM in Figure 2B.

C) Tweedie GLM for deer mouse density ~ diet competition + productivity at web level.

D) Same model at site level.

E) Binomial GLM for Figure 5B.

F) Binomial GLM for Figure 5C.

G) Tweedie GLM for Figure 5D.

H) Tweedie GLM for Figure 5E.

SI Tables

Table S1. Species 2-letter abbreviations used in figures.

Species Code	Species
ai	*Ammospermophilus interpres*
ch	*Chaetodipus hispidus*
ci	*Chaetodipus intermedius*
dm	*Dipodomys merriami*
do	*Dipodomys ordii*
ml	*Microtus longicaudus*
mm	*Microtus mexicanus*
mn	*Microtus montanus*
mp	*Microtus pennsylvanicus*
mu	*Mus musculus*
nc	*Neotoma cinerea*
ni	*Neotoma micropus*
nl	*Neotoma lepida*
nm	*Neotoma mexicana*
ns	*Neotoma stephensi*
oa	*Onychomys arenicola*
ol	*Onychomys leucogaster*
pb	*Peromyscus boylii*
pe	*Peromyscus eremicus*
pf	*Perognathus flavescens*
pl	*Peromyscus leucopus*
pm	*Peromyscus maniculatus*
pn	*Peromyscus nasutus*
pt	*Peromyscus truei*
pv	*Perognathus flavus*
rg	*Reithrodontomys megalotis*
ro	*Reithrodontomys montanus*
sh	*Sigmodon hispidus*
sp	*Spermophilus spilosoma*
sv	*Spermophilus variegatus*
td	*Tamias dorsalis*
tf	*Tamias rufus*
tm	*Tamias minimus*
tq	*Tamias quadrivittatus*

Table S2. Results from binomial GLM modeling SNV prevalence as a function of rodent diversity in Figure 2A. Models included an indicator variable ("in Luis et al. 2018") identifying webs previously analyzed in Luis et al. (2018), allowing direct comparison between the original nine webs and the additional webs included in the expanded dataset. The variable and interaction were important, suggesting a different diversity-disease relationship between those webs included vs not included in the previous study. See Fig. S9a for diagnostic plot.

	Estimate	Std. Error	z-value	p-value
Intercept	-6.007	1.259	-4.771	<0.001
Rodent Diversity	0.582	0.441	1.321	0.187
in Luis et al. 2018	5.161	1.382	3.734	<0.001
Rodent Diversity : in Luis et al. 2018	-1.153	0.534	-2.158	0.031

Table S3. Results from Tweedie GLM modeling infected host density as a function of rodent diversity in Figure 2B. Models included an indicator variable ("in Luis et al. 2018") identifying webs previously analyzed in Luis et al. (2018), allowing direct comparison between the original nine webs and the additional webs included in the expanded dataset. The variable and interaction were important, suggesting a different diversity-disease relationship between those webs included vs not included in the previous study. See Fig. S9b for diagnostic plot.

	Estimate	Std. Error	z-value	p-value
Intercept	-5.964	0.241	-24.782	<0.001
Rodent Diversity	1.254	0.088	14.275	<0.001
in Luis et al. 2018	10.276	0.287	35.785	<0.001
Rodent Diversity : in Luis et al. 2018	-2.785	0.122	-22.793	<0.001

Table S4. Summary of Redundancy Analysis results including eigenvalues and explanatory power of constrained axes (comprised of environmental variables) labeled as 'RDA' and unconstrained axes labeled as 'PC' (additional correlations between species abundances).

	Eigenvalue	Proportion Explained	Cumulative Proportion
RDA1	0.187	0.337	0.337
RDA2	0.129	0.232	0.569
RDA3	0.076	0.136	0.705
RDA4	0.037	0.067	0.772
RDA5	0.024	0.043	0.815
RDA6	0.011	0.020	0.835
RDA7	0.003	0.006	0.841
RDA8	0.001	0.002	0.843
PC1	0.035	0.063	0.906
PC2	0.014	0.026	0.932
PC3	0.010	0.019	0.951
PC4	0.008	0.015	0.966
PC5	0.006	0.010	0.976
PC6	0.004	0.007	0.982
PC7	0.003	0.006	0.988
PC8	0.002	0.004	0.992
PC9	0.001	0.002	0.994
PC10	0.001	0.002	0.996
PC11	0.001	0.001	0.998
PC12	0.001	0.001	0.999
PC13	0.000	0.001	0.999
PC14	0.000	0.000	1.000
PC15	0.000	0.000	1.000

Table S5. Significance of permutation tests on predictor variables from the RDA, with 1000 permutations.

	Df	Variance	F	p-value
temperature min	1	0.15	26.30	0.0010
temperature max	1	0.06	10.62	0.0010
precipitation	1	0.08	13.52	0.0010
snow	1	0.05	7.95	0.0010
elevation	1	0.02	4.22	0.0040
productivity	1	0.04	7.04	0.0010
tree cover	1	0.05	8.64	0.0010
bare ground	1	0.01	2.20	0.0420
Residual	15	0.09		

Table S6. Significance of permutation tests on the canonical axes from the RDA, with 1000 permutations.

	Df	Variance	F	p-value
RDA1	1	0.19	32.14	0.0010
RDA2	1	0.13	22.18	0.0010
RDA3	1	0.08	13.00	0.0010
RDA4	1	0.04	6.37	0.0010
RDA5	1	0.02	4.11	0.0130
RDA6	1	0.01	1.91	0.3630
RDA7	1	0.00	0.56	0.9660
RDA8	1	0.00	0.22	0.9940
Residual	15	0.09		

Table S7. Model comparisons of biotic regressions with web as the replicate. AIC weight 1 shows the AIC weights when considering only the models with traits. AIC weight 2 shows weights when adding productivity to the best trait model.

model	AIC	AIC weight 1	AIC weight 2
Diet Competition + Productivity	5064.0062	–	1.0000
Diet Competition	5112.3644	1.0000	0.0000
Phylogenetic Competition	5417.8715	0.0000	0.0000
Simpson's diversity	5424.2519	0.0000	0.0000
Mass Competition	5433.5978	0.0000	0.0000
Activity Competition	5433.9948	0.0000	0.0000

Table S8. Results of best GLM for deer mouse abundance (web as replicate) using functional traits. See Fig S9c for diagnostic plot.

	Estimate	Std. Error	z-value	p-value
Intercept	3.1641	0.0738	42.8954	<0.0001
Diet Competition	-0.1136	0.0047	-24.1484	<0.0001
Productivity	0.3506	0.0537	6.5320	<0.0001

Table S9. Model comparisons of biotic regressions with site as the replicate, using means of webs per site. AIC weight 1 shows the AIC weights when considering only the models with traits. AIC weight 2 shows weights when adding productivity to the best trait model.

model	AIC	AIC weight 1	AIC weight 2
Diet Competition + Productivity	2025.7667	–	1.0000
Diet Competition	2052.0460	1.0000	0.0000
Mass Competition	2163.8456	0.0000	0.0000
Activity Competition	2173.9540	0.0000	0.0000
Phylogenetic Competition	2198.3220	0.0000	0.0000
Simpson's diversity	2245.9692	0.0000	0.0000

Table S10. Results of best GLM for deer mouse abundance (site as replicate) using functional traits. See Fig S9d for diagnostic plot.

	Estimate	Std. Error	z-value	p-value
Intercept	3.2591	0.0938	34.73	<0.0001
Diet Competition	-0.1184	0.0062	-19.22	<0.0001
Productivity	0.3180	0.0641	4.97	<0.0001

Table S11. Summary of nestedness and co-occurrence metrics based on rodent presence–absence data. The table reports the observed value of each metric (Statistic), the mean of the null distribution (from 999 simulations using the "quasiswap" algorithm), and the standardized effect size (SES). P-value is two-sided, where the alternative hypothesis is significantly nested. Matrix temperature reflects deviation from perfect nestedness (lower values indicate stronger nestedness). C-score = co-occurrence metric based on checkerboard units; lower values than null model indicate anti-nestedness. NODF = Nestedness metric based on Overlap and Decreasing Fill; lower values than null indicate anti-nestedness.

Method	Statistic	Mean Null	SES	P-value
Matrix Temperature	34.35	32.51	1.25	0.213
C-score	10.16	24.13	-20.64	0.001
NODF	47.92	51.94	-0.65	0.001

Table S12. Standardized estimates, standard errors (SE) and p-values for the paths in the structural equation model (SEM) presented in the main text (Fig. 4). AIC = 199.39.

Outcome		Predictor	Std Estimate	SE	p-value
diet competitors	~	abiotic variables	0.954	0.118	<0.001
diet competitors	~	productivity	0.763	0.173	<0.001
productivity	~	abiotic variables	-0.607	0.117	<0.001
deer mouse abundance	~	diet competitors	-0.695	0.117	<0.001
deer mouse abundance	~	productivity	0.486	0.135	<0.001
prevalence	~	deer mouse abundance	0.869	0.048	<0.001

Table S13. Standardized estimates, standard errors (SE) and p-values for the paths in the SEM including a direct effect of abiotic variables on deer mouse abundance. There is not support for adding the additional path. AIC = 201.39.

Outcome		Predictor	Std Estimate	SE	p-value
diet competitors	~	abiotic variables	0.954	0.118	<0.001
diet competitors	~	productivity	0.763	0.173	<0.001
productivity	~	abiotic variables	-0.607	0.117	<0.001
deer mouse abundance	~	diet competitors	-0.694	0.198	<0.001
deer mouse abundance	~	productivity	0.486	0.229	0.034
deer mouse abundance	~	abiotic variables	-0.001	0.257	0.997
prevalence	~	deer mouse abundance	0.869	0.048	<0.001

Table S14. Standardized estimates, standard errors (SE) and p-values for the paths in the SEM including a link from rodent diversity to deer mouse abundance. There is not strong support for the additional path. AIC = 200.11.

Outcome		Predictor	Std Estimate	SE	p-value
diet competitors	~	abiotic variables	0.954	0.118	<0.001
diet competitors	~	productivity	0.763	0.173	<0.001
productivity	~	abiotic variables	-0.607	0.117	<0.001
deer mouse abundance	~	diet competitors	-0.778	0.095	<0.001
deer mouse abundance	~	productivity	0.345	0.124	0.006
deer mouse abundance	~	rodent diversity	0.230	0.114	0.044
prevalence	~	deer mouse abundance	0.886	0.042	<0.001

Table S15. Binomial GLM model comparisons for Figure 5B, examining how Simpson's diversity index and grouping of clusters affects SNV prevalence. There was support for grouping the blue community cluster 4 with clusters 1 and 2 as part of the 'Dilution group'. There was also support for the additive (+) model over the model with an interaction (*) term.

model	AIC	AIC weight
Simpson's D + Grouping 1,2,4	69.19	0.57
Simpson's D * Grouping 1,2,4	71.19	0.21
Simpson's D * Grouping 1,2	71.81	0.15
Simpson's D + Grouping 1,2	73.28	0.07

Table S16. Results of the binomial GLM for how rodent diversity affects SNV prevalence, with sites grouped by dilution effect or non-dilution effect clusters, represented in Figure 5B. See Fig. S9e for diagnostic plot.

	Estimate	Std. Error	z-value	p-value
intercept	-0.753	0.473	-1.591	0.112
rodent diversity	-0.699	0.231	-3.026	0.002
non-dilution group	-3.348	0.766	-4.369	<0.001

Table S17. Partitioning of beta diversity into turnover and nestedness components based on Sørensen dissimilarity. Total dissimilarity (β_{SOR}) was divided into the turnover component (β_{SIM}), representing species replacement among sites, and the nestedness component (β_{SNE}) representing species loss or gain without replacement. Results indicate that most variation in community composition was driven by turnover rather than nestedness.

	β_{SOR}	β_{SIM}	β_{SNE}
All Communities	0.859	0.800	0.058
Dilution Effect Group	0.786	0.696	0.090
Non-Dilution Effect Group	0.708	0.614	0.094

Table S18. Summary of nestedness and co-occurrence metrics based on rodent presence–absence data for the ‘Dilution effect group’ and ‘Non-dilution effect group’. The table reports the observed value of each metric (Statistic), the mean of the null distribution (from 999 simulations using the "quasiswap" algorithm), and the standardized effect size (SES). P-value is two-sided, where the alternative hypothesis is significantly nested. Matrix temperature reflects deviation from perfect nestedness (lower values indicate stronger nestedness). C-score = co-occurrence metric based on checkerboard units; lower values than null model indicate anti-nestedness. NODF = Nestedness metric based on Overlap and Decreasing Fill; lower values than null indicate anti-nestedness.

Method	Statistic	Mean Null	SES	P-value
Dilution Effect Group				
Matrix Temperature	23.75	24.73	-0.84	0.401
C-score	2.71	6.43	-12.61	0.001
NODF	35.91	43.49	-0.65	0.203
Non-Dilution Effect Group				
Matrix Temperature	25.49	26.04	-0.83	0.393
C-score	1.71	5.14	-12.09	0.001
NODF	32.99	43.23	-0.68	0.059

Table S19. Results of the binomial GLM for how deer mouse density affects SNV prevalence, with sites grouped by dilution effect or non-dilution effect, represented in Figure 5C. See Fig. S9f for diagnostic plot.

	Estimate	Std. Error	z-value	p-value
intercept	-2.923	0.247	-11.832	<0.001
deer mouse density	0.037	0.008	4.336	<0.001
non-dilution group	-2.666	0.789	-3.378	0.001

Table S20. Results of the Tweedie GLM for how rodent diversity affects deer mouse density, with sites grouped by dilution effect or non-dilution effect, represented in Figure 5D. See Fig. S9g for diagnostic plot.

	Estimate	Std. Error	z-value	p-value
intercept	3.843	0.099	38.673	<0.001
rodent diversity	-0.521	0.041	-12.676	<0.001
non-dilution group	-0.445	0.367	-1.212	0.226
rodent diversity : non-dilution group	-1.812	0.171	-10.621	<0.001

Table S21. Results of the Tweedie GLM for how rodent diversity affects diet competition, with sites grouped by dilution effect or non-dilution effect, represented in Figure 5E. Diet competition was calculated as the sum of competitor densities times their similarity in diet overlap with deer mice. See Fig. S9h for diagnostic plot.

	Estimate	Std. Error	z-value	p-value
intercept	0.754	0.075	10.03	<0.001
rodent diversity	0.598	0.031	19.55	<0.001
non-dilution group	2.959	0.131	22.58	<0.001
rodent diversity : non-dilution group	-0.982	0.057	-17.08	<0.001

Legends for Datasets published on Zenodo

https://doi.org/10.5281/zenodo.18744824

WebSpeciesData.csv

Dataset of average species abundances (columns) per trapping web (rows). Two-letter codes corresponding to species names are provided in "SpeciesTraitData.csv" dataset and SI Appendix Table S1. Additionally contains number of months the web was trapped ("n_months"), average rodent diversity by inverse Simpson's D diversity index ("invSimpson"), average SNV prevalence among deer mice ("prevalence"), average infected deer mouse density ("IHD"), and whether or not the site was included in Luis et al. (2018) modeling study ("in2018pub").

EnvironmentalData.csv

Average environmental conditions per web, including daily temperature minima ("tmin") and maxima ("tmax") (°C), daily total precipitation in mm/day (sum of all forms converted to water, "precip"), snow-water equivalent (km/m2) – a measurement of the amount of water contained within the snowpack ("swe" referred to as "snow" in the main text), and elevation. Additionally, from the Rangeland Analysis Platform (rangelands.app), new herbaceous above ground biomass over each 16-day period ("biomass" referred to as "productivity" in the main text) which is then summed to an annual total and averaged, average percent tree cover, and average percent bare ground.

SpeciesTraitData.csv

Rodent species 2-letter codes and species trait data from EltonTraits 1.0 database (Wilman *et al.* 2014) including average adult body mass, whether nocturnal, and the proportion of their diet belonging to the following categories: invertebrates, endotherms, ectotherms, fish, vertebrates (general), scavenging, fruit, nectar, seed, and plant.

PhyloTree.txt

Phylogenetic tree of rodent species in the dataset, taken from the Bininda-Emonds et al. (2007) mammalian supertree.

Code.R

R code for analyses.

References

Allred, B.W., Bestelmeyer, B.T., Boyd, C.S., Brown, C., Davies, K.W., Duniway, M.C., *et al.* (2021). Improving Landsat predictions of rangeland fractional cover with multitask learning and uncertainty. *Methods Ecol Evol*, 12, 841–849.

Bagamian, K.H., Towner, J.S., Mills, J.N. & Kuenzi, A.J. (2013). Increased detection of Sin Nombre hantavirus RNA in antibody-positive deer mice from Montana, USA: Evidence of male bias in RNA viremia. *Viruses*, 5, 2320–2328.

Bininda-Emonds, O.R., Cardillo, M., Jones, K.E., MacPhee, R.D., Beck, R.M., Grenyer, R., *et al.* (2007). The delayed rise of present-day mammals. *Nature*, 446, 507.

Borcard, D., Gillet, F. & Legendre, P. (2018). *Numerical ecology with R*. Springer.

Carver, S., Mills, J.N., Parmenter, C.A., Parmenter, R.R., Richardson, K.S., Harris, R.L., *et al.* (2015). Toward a mechanistic understanding of environmentally forced zoonotic disease emergence: Sin Nombre Hantavirus. *Bioscience*, 65, 651–666.

Gotelli, N.J. (1998). *A primer of ecology*, 4th edn Sunderland. MA: Sinauer Associates.

Hartig, F. (2024). DHARMa: Residual Diagnostics for Hierarchical (Multi-Level / Mixed) Regression Models. *R package version 0.4.7. https://CRAN.R-project.org/package=DHARMa*.

Hufkens, K., Basler, D., Milliman, T., Melaas, E.K. & Richardson, A.D. (2018). An integrated phenology modelling framework in R. *Methods Ecol Evol*, 9.

Jones, M.O., Robinson, N.P., Naugle, D.E., Maestas, J.D., Reeves, M.C., Lankston, R.W., *et al.* (2021). Annual and 16-day rangeland production estimates for the western United States. *Rangel Ecol Manag*, 77, 112–117.

Keddy, P.A. & Laughlin, D.C. (2021). *A framework for community ecology: species pools, filters and traits*. Cambridge University Press.

Kéry, M. & Schaub, M. (2012). *Bayesian Population Analysis Using WinBUGS: A Hierarchical Perspective*. Academic Press.

Luis, A.D., Douglass, R.J., Mills, J.N. & Bjørnstad, O.N. (2015). Environmental fluctuations lead to predictability in Sin Nombre hantavirus outbreaks. *Ecology*, 96, 1691–1701.

Luis, A.D., Kuenzi, A.J. & Mills, J.N. (2018). Species diversity concurrently dilutes and amplifies transmission in a zoonotic host-pathogen system through competing mechanisms. *Proceedings of the National Academy of Sciences*, 115, 7979–7984.

McGillycuddy, M., Warton, D.I., Popovic, G. & Bolker, B.M. (2025). Parsimoniously Fitting Large Multivariate Random Effects in **glmmTMB**. *J Stat Softw*, 112, 1–19.

Mills, J.N., Yates, T.L., Ksiazek, T.G., Peters, C.J. & Childs, J.E. (1999). Long-term studies of hantavirus reservoir populations in the Southwestern United States: rationale, potential, and methods. *Emerg Infect Dis*, 5, 95–101.

Myhrvold, N.P., Baldridge, E., Chan, B., Sivam, D., Freeman, D.L. & Ernest, S.K.M. (2015). An amniote life-history database to perform comparative analyses with birds, mammals, and reptiles: Ecological Archives E096-269. *Ecology*, 96, 3109.

Oksanen, J., Simpson, G.L., Blanchet, F.G., Kindt, R., Legendre, P., Minchin, P.R., *et al.* (2022). vegan: Community Ecology Package.

Paradis, E. & Schliep, K. (2019). ape 5.0: an environment for modern phylogenetics and evolutionary analyses in R. *Bioinformatics*, 35, 526–528.

Rosseel, Y. (2012). Journal of Statistical Software lavaan: An R Package for Structural Equation Modeling. *J Stat Softw*, 48.

Stahel, W.A. (2002). *Statistische Datenanalyse: Eine Einführung für Naturwissenschaftler*. 4th edn. Vieweg, Braunschweig.

Su, Y.-S. & Masanao Yajima. (2021). R2jags: Using R to Run "JAGS."

Thornton, M.M., Shrestha, R., Wei, Y., Thornton, P.E., Kao, S.C. & Wilson, B.E. (2022). Daymet: Daily Surface Weather Data on a 1-km Grid for North America, Version 4 R1. ORNL DAAC, Oak Ridge, Tennessee, USA.

Warton, D.I. & Hui, F.K.C. (2011). The arcsine is asinine: The analysis of proportions in ecology. *Ecology*, 92.

Whitmer, S.L.M., Whitesell, A., Mobley, M., Talundzic, E., Shedroff, E., Cossaboom, C.M., *et al.* (2024). Human Orthohantavirus disease prevalence and genotype distribution in the U.S., 2008–2020: a retrospective observational study. *The Lancet Regional Health - Americas*, 37.

Wilman, H., Belmaker, J., Simpson, J., de la Rosa, C., Rivadeneira, M.M. & Jetz, W. (2014). EltonTraits 1.0: Species-level foraging attributes of the world's birds and mammals: Ecological Archives E095-178. *Ecology*, 95, 2027.

Yates, T., Mills, J.N., Parmenter, C.A. & Ksiazek, T.G. (2002). The ecology and evolutionary history of an emergent disease: hantavirus pulmonary syndrome. *Bioscience*, 52, 989–998.
